# The quantity-quality transition in the value of expanding wind and solar power generation

**DOI:** 10.1016/j.isci.2022.104140

**Published:** 2022-03-22

**Authors:** Enrico G.A. Antonini, Tyler H. Ruggles, David J. Farnham, Ken Caldeira

**Affiliations:** 1 Carnegie Institution for Science, Department of Global Ecology, Stanford, CA 94305, USA; 2 Breakthrough Energy, Kirkland, WA 98033, USA

**Keywords:** Energy resources, Energy policy, Energy sustainability, Energy flexibility

## Abstract

Wind and solar photovoltaic generators are projected to play important roles in achieving a net-zero-carbon electricity system that meets current and future energy needs. Here, we show potential advantages of long-term site planning of wind and solar power plants in deeply decarbonized electricity systems using a macro-scale energy model. With weak carbon emission constraints and substantial amounts of flexible electricity sources on the grid (e.g., dispatchable power), relatively high value is placed on sites with high capacity factors because the added wind or solar capacity can efficiently substitute for running natural gas power plants. With strict carbon emission constraints, relatively high value is placed on sites with high correlation with residual demand because resource complementarity can efficiently compensate for lower system flexibility. Our results suggest that decisions regarding long-term wind and solar farm siting may benefit from consideration of the spatial and temporal evolution of mismatches in electricity demand and generation capacity.

## Highlights

• We show potential advantages of long-term planning of wind and solar power plants
• In weak decarbonization, wind and solar resource quantity governs site decisions
• In deep decarbonization, optimal sites have higher correlation with residual demand
• Siting decisions made with foresight can lead to more efficient asset allocation

## Graphical abstract

**Figure:**
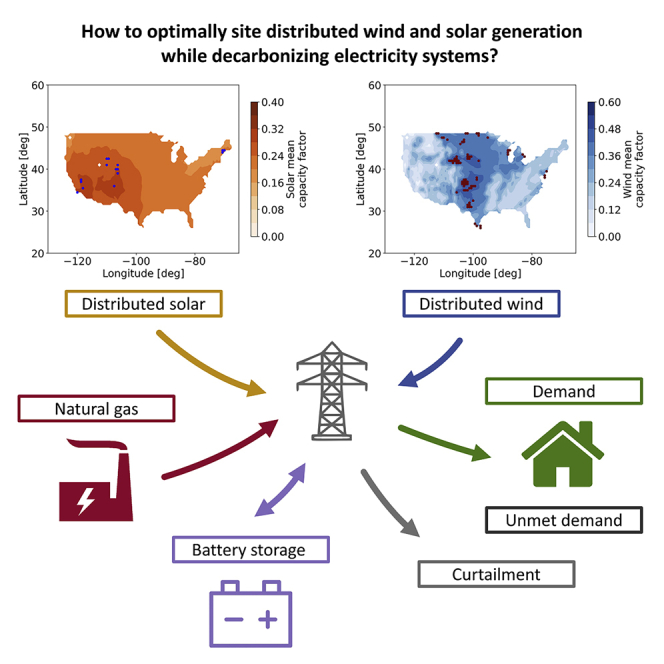

## Introduction

Wind and solar power are likely to play important roles in any successful transition to a future net-zero emissions electricity system. The 2020 share of electricity generated from wind was globally 6.1%, and 3.3% was from solar photovoltaics (Our World In Data, 2021). In recent years, these technologies have been among the fastest growing (Enerdata, 2020). The continued fall in costs of wind and solar technologies (International Renewable Energy Agency (IRENA), 2020) along with ambitious climate policies in the E.U., the U.S., China, and India, among others (European Energy Commission, 2019; International Renewable Energy Agency (IRENA), 2015; Sierra Club, 2020), contributed to recent increases in wind and solar capacities and power generation. However, meeting targets for net-zero emissions electricity systems will likely require substantial wind and solar capacity additions.

Adding higher shares of wind and solar generation to a power system introduces the challenge of managing greater generation variability. Wind and solar resources are highly variable in space and time and not always available in quantities needed to meet electricity demand (Bird et al., 2013; Lund et al., 2015; Rinaldi et al., 2021). Although wind and solar resources in certain regions have some degree of complementarity that can help mitigate and smooth variability (Li et al., 2009; Miglietta et al., 2017), reliable power systems based primarily on variable energy sources require effective grid management, backup power systems, and energy storage capacity (Aghaei and Alizadeh, 2013; Huber et al., 2014; Su and El Gamal, 2013). A solution enabled by energy storage is temporally shifting the variable electricity generation: energy is stored in times when it would be otherwise curtailed and used in times when the variable electricity generation is lower than current demand (Kroposki et al., 2017). Energy can be stored both for short duration (e.g., in lithium-ion batteries or flywheels), when quick, intra-daily demand compensation is needed (Yang et al., 2018), and long duration (e.g., in power-to-gas-to-power, pumped hydro, or compressed air storage), when inter-day, inter-season, or even multi-year storage is needed (Blanco and Faaij, 2018; Dowling et al., 2020). In addition to storage, low- or zero-carbon firm generators (e.g., nuclear) could potentially contribute to lowering the overall cost of decarbonized systems (Sepulveda et al., 2018; Yuan et al., 2020). Despite these challenges faced by utilities and system operators with high penetration of variable generation, a variety of operational and technical solutions exist to add and integrate wind and solar generation that could be implemented in future years (Antonini et al., 2021; Denholm and Hand, 2011; Diao et al., 2010; Elliott, 2016; Frew et al., 2016; Gielen et al., 2019; Jenkins et al., 2018; Ruggles et al., 2021).

A crucial question that arises when planning for new power generation facilities at the system level is determining where to site new generation. Sites for power generation tend to be highly stable in space and time, due to factors such as capital investment in plant and facilities, electricity transmission, permitting constraints, access to water, etc. The issue of siting is particularly important for wind and solar installations because their economic viability depends largely on the availability of resources that are highly variable in space and time. Thus far, geophysical considerations governing site selection of solar installations are mostly solar irradiance and equivalent sun hours (Arán Carrión et al., 2008; Ramachandra and Shruthi, 2007), whereas for wind power plants, the primary geophysical consideration is mean wind power density at the turbine hub height (Becker and Thrän, 2018; Cetinay et al., 2017). For wind power plants, system-level planning is then followed by turbine micro-siting to determine the positions of the individual turbines to minimize wake losses (Antonini et al., 2016, 2018a, 2018b, 2019, 2020; Dhoot et al., 2021). Methods for optimal siting in a distributed network have been developed to consider additional criteria related to environmental, economic, social, and technical aspects (Georgiou and Skarlatos, 2016; Prakash and Khatod, 2016; Tegou et al., 2010). Particular attention has been directed to the integration of distributed generation into the electrical grid by looking, for example, at line loss reduction, proximity to existing grid interconnects, or increased system voltage profile (Al Abri et al., 2013; Kayal and Chanda, 2013). However, these electrical integration studies have addressed local, small-scale distribution networks (Ghosh et al., 2010; Sadeghian et al., 2017). At large scale, energy systems planning tools have been extensively used to evaluate the distributions of wind and solar developments, either to meet certain generation thresholds (Jerez et al., 2015) or to integrate them with the electric grid and demand profiles (Bussar et al., 2016; Fürsch et al., 2013). However, the drivers governing their optimal locations have not been systematically examined, especially for deeply decarbonized systems with a high penetration of wind and solar generation.

Here, we show potential advantages of long-term planning when relying on distributed wind and solar power to decarbonize an electricity system. We evaluate optimal siting of distributed wind and solar generation in combination with energy storage and natural gas generation with increasingly strict carbon emissions limits. We illustrate in an idealized setting how siting decisions made with foresight can lead to more efficient asset allocation. Real-world siting decisions would require detailed analysis of both current conditions and anticipated future conditions, and an understanding of how net present value depends on some balance of current value and expected future value. With application to the contiguous U.S. (CONUS), we use hourly time-averaged wind and solar resource data and electricity demand data as inputs to a macro-scale energy model (Levi et al., 2019). The spatial distribution of wind and solar generation is evaluated based on the subdivision of CONUS into the grid cells of a weather reanalysis dataset. For each cell, we calculate hourly capacity factor time series of wind and solar. The macro-scale energy model allows us to evaluate least-cost solutions with installed capacities and dispatch schedules at each location that, in combination with energy storage and natural gas, meet the aggregated hourly demand time series (see STAR Methods section, Figure 4, and Table 1 for additional details). In evaluating capacities and dispatch schedules, we consider increasingly strict carbon emissions limits from natural gas to ultimately reach a decarbonized electricity system. The optimization to meet those emissions constraints is run with both multi-step and single-step optimizations to assess the impact of a short vs. long-term system planning strategy. Our analysis is stylized and aims to highlight differences between multi-step and single-step approaches to decarbonizing electricity systems. Our results are not intended to guide real-world siting decisions, which would need to take into account more factors than considered here. In the supplemental information, we show application to a smaller region, i.e., Texas, operated by the Electric Reliability Council of Texas (ERCOT). This application allows us to assess any differences between continental and regional scales and highlight any potential advantage of one versus the other. To complete and further strengthen our analysis, for ERCOT, we also consider a case with two additional technologies, i.e., nuclear generation and power-to-gas-to-power (PGP) storage, in combination with battery storage and wind, solar, and natural gas generation. We also show the results of this additional analysis in the supplemental information.

## Results

The results are organized as follows: we first show a high-level analysis of the system architectures and costs resulting from increasingly strict carbon emissions limits for both multi-step and single-step optimizations. We then analyze statistical correlations between the wind and solar capacity factors of the selected locations and demand profiles for increasing carbon emission reductions. Lastly, we show the spatial distribution of the selected locations for wind and solar installations.

**Figure 1:**
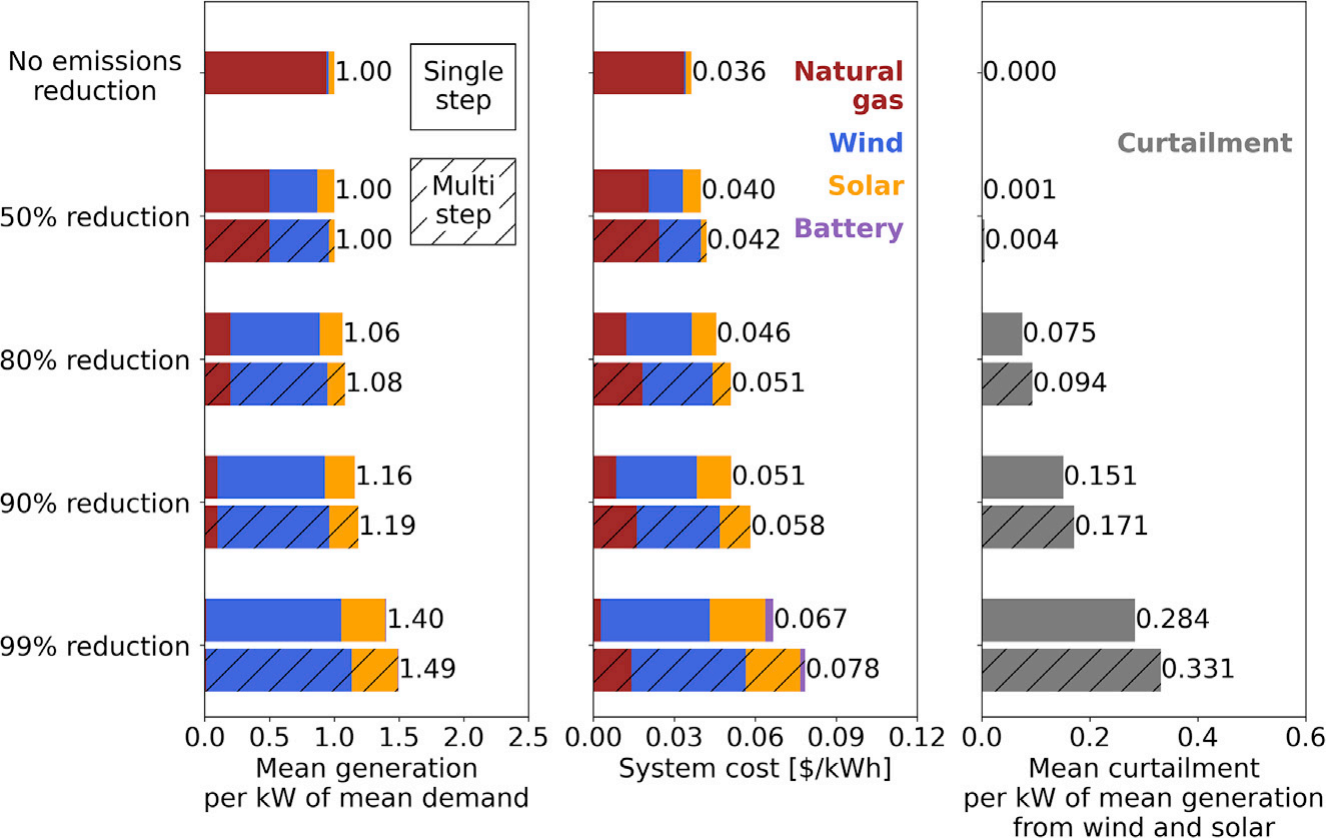
Mean generation, system-level cost, and mean curtailment for increasingly strict carbon emissions limits resulting from both multi-step and single-step optimizations The mean electricity demand is approximately equal to 460 GW.

In Figure 1, per kW of mean electricity demand, we show mean generation, system-level cost, and mean curtailment of wind and solar generation for increasingly strict carbon emissions limits resulting from both multi-step and single-step optimizations. The mean electricity demand is approximately equal to 460 GW. This means, for example, that a mean generation of 1.5 times greater than the mean demand corresponds to a mean generation of about 690 GW (∼150% of the mean demand). Some of the generation by wind and solar may be curtailed. For example, in the 99%-emissions-reduction multi-step case, about 33% of the mean power generated by wind and solar is curtailed, which corresponds to a mean value of about 230 GW (or about 2000 TWh in terms of annual energy). In all cases, at least 99.999% of demand is met, and penalty costs for unmet demand are always less than 0.2% of the total system cost. With no emission limits, the electricity is generated almost entirely by natural gas with wind and solar accounting for 1.8% and 4.2%, respectively, of the total mean generation. Because of the relatively low cost of natural gas, this system that relies heavily on natural gas is the one with the lowest cost at 0.036 $/kWh. The high level of flexibility provided by natural gas combined with the low share of wind and solar electricity generation leads to no electricity being curtailed.

As we impose reductions of carbon emissions, multi-step and single-step optimizations result in different systems. As emissions decrease, both approaches lead to an increase in mean generation, system cost, and mean curtailment. Note that curtailment applies only to wind and solar generation. The multi-step optimization results in larger increases in mean generation, system cost, and mean curtailment compared to the single-step optimization. For example, for an 80% reduction in CO_2_, we have an increase in total mean generation with respect to the no-emission-reduction case of 8.3% and 6.5% for multi-step and single-step optimizations, respectively. A consistent increase over the no-emission-reduction case is observed for the system cost, where multi-step and single-step optimizations lead to 40% and 25% higher energy costs than the no-emission-reduction case, respectively. A 99% reduction in carbon emissions results in a system with the highest mean generation, system costs, and mean curtailment. The multi-step optimization leads to a system that has 6.7% more mean generation and 18% higher cost than a single-step optimization. If we were to consider an annual electricity consumption of about 3800 TWh in 2020 in the U.S. (U.S. Energy Information Administration (EIA), 2021), the difference in system cost between the multi-step and single-step optimizations for the 99%-emissions-reduction case can be estimated to be about 42 billion dollars per year (annual electricity consumption multiplied by the difference in system costs).

The system flexibility originally provided by natural gas generation in the no-emissions-reduction case is not provided by a large share of battery storage in cases with high amounts of emission reduction. The mean generation of battery storage is 0.6% and 0.2% of the total at a 99% emissions reduction for multi-step and single-step optimizations, respectively. In general, these simulations suggest that battery storage technology is used to fill short-term gaps between variable wind or solar generation and hourly demand. Our simulations indicate that battery storage is not cheap enough, at today’s cost, to be an economically competitive solution for seasonal storage in deeply decarbonized energy systems. These findings are consistent with a previous study (Tong et al., 2020) that looked at different cost reductions for energy storage. Energy storage with capacity costs consistent with battery technology was mainly used for short periods of time while energy storage with capacity costs consistent with compressed air or pumped hydroelectricity technologies was used for seasonal energy storage. Consequently, the missing system flexibility for deep decarbonization scenarios results in a higher percentage of wind and solar energy being curtailed. At 99% emissions reduction, curtailment is applied to about 33% and 28% of the mean wind and solar generation for multi-step and single-step optimizations, respectively.

**Figure 2:**
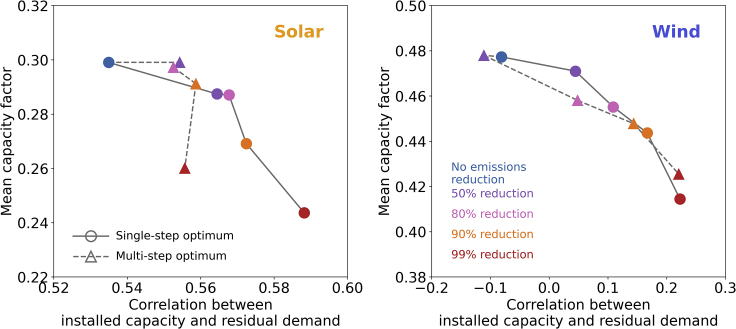
Mean capacity factors of wind and solar installations versus the correlation between the capacity factor time series and the residual demand for different emissions reduction targets The left panel shows results for the solar installations, while the right panel for wind. For increasingly strict carbon emissions limits, mean capacity factors generally decrease and correlations with residual demand increase.

We also perform a statistical analysis of the wind and solar capacity factors of the selected locations for various emission reduction targets. Specifically, we analyze how the mean and standard deviation of capacity factors of the chosen locations vary for increasingly strict carbon emissions limits. We calculate the correlation of wind and solar capacity factor time series with the demand time series for different emissions reduction cases. We also calculate the correlation of wind and solar capacity factor time series with the residual demand time series. The residual demand is calculated as the demand time series minus the aggregate solar electricity generation time series when the correlation is evaluated with the wind capacity factor time series, or minus the aggregate wind electricity generation time series when the correlation is evaluated with the solar capacity factor time series. This quantity is useful to evaluate the degree of complimentary between the wind and solar resources. We lastly calculate correlations between wind and solar capacity factor time series and the demand time series when smoothing filters are applied in the time domain. Specifically, we apply a 24-hour (daily) moving average, with which we generate inter-daily and sub-daily correlations—the latter calculated by subtracting the 24-hour moving average from the original time series—and a 30-day (monthly) moving average. These moving averages are useful to disentangle the contribution from each of the time series components (sub-daily, inter-daily, and inter-monthly) and evaluate any statistical relevance of one versus the other. Extensive results from this statistical analysis are reported in the supplemental information. Of all the quantities that we evaluated, we noted that only two showed a noticeable change in the mean value for increasingly strict emissions reductions. We convey these main findings in the phase plots of Figure 2.

In Figure 2, we plot the changes in mean capacity factors of wind and solar installations versus the correlation between the capacity factor time series and the residual demand for different emissions reduction targets. The mean capacity factor of the chosen locations shows a clear decrease as emissions reductions are applied; this is true both for single-step and multi-step optimizations. For wind in the single-step optimization, the mean capacity factor decreases from 0.48 in the no-emissions-reduction case to 0.41 in the 99%-emissions-reduction case. For solar in the single-step optimization, the mean capacity factor decreases from 0.30 in the no-emissions-reduction case to about 0.24 in the 99%-emissions-reduction case. In the multi-step optimization, the mean capacity factors with a 99% emissions reduction decrease to 0.42 for wind and 0.26 for solar. This trend helps better explain the higher system cost that we observed earlier: as emissions limits become stricter and natural gas phases out, the system becomes more reliant on wind and solar generation, which have higher fixed costs than natural gas as well as lower capacity factors. As emissions reductions are enforced, the correlation between the capacity factor time series and the residual demand increases. In the single-step optimization, correlations with residual demand increase from about −0.07 for wind and 0.53 for solar with no emissions limits, to about 0.22 for wind and 0.59 for solar with a 99% emissions reduction. In the multi-step optimization, the correlations with a 99% emissions reduction increase to about 0.22 for wind and 0.55 for solar. This indicates that as emissions limits become stricter and natural gas phases out, optimal locations have a higher degree of complementarity to compensate for lower system flexibility. Also, we note a larger standard deviation and range of the correlation values for stricter emissions limits (see the supplemental information). This indicates that as the system becomes less flexible, locations with different capacity factor time series help fully meet all periods of electricity demand.

In general, with a single-step optimization, mean capacity factors decrease and correlation with residual demand increases for increasingly strict carbon emissions limits. This behavior was present in the vast majority of cases; however, we note one case—i.e., solar mean capacity factor in the year 2017, reported in the supplemental information—where for increasingly strict carbon emissions limits, we had a decrease in mean capacity factor but a nearly constant, and sometimes decreasing, correlation with residual demand. With a multi-step optimization, we observe similar, even if more irregular, trends in capacity factor and correlation, which we attribute to system sub-optimality as described below. Recall that the single-step solutions assume that the system can be built from scratch for a given emissions reduction target, thus the solutions each represent a global optimum. The multi-step solutions, instead, start from an infrastructure built for a weaker emissions reduction target. They therefore inherit some built capacity that does not necessarily coincide with the global optimum of the single-step solution for the same emissions. This sub-optimality helps explain why the correlation often decreases in the multi-step solutions as we enforce stricter emissions reduction targets. While the system is flexible thanks to the natural gas generation, places with the highest capacity factor are more likely to be selected, but as the system loses flexibility, correlation with times when generation is insufficient to meet demand becomes more important. Note also that in most emission-reduction cases, a multi-step solution results in slightly higher average capacity factors for installed wind and solar than a single-step solution, albeit a more costly system.

In the supplemental information, we conduct a similar analysis for ERCOT. The results are overall consistent with CONUS in terms of mean generation, system cost, and mean curtailment. From the statistical analysis, however, we note a substantial difference: because solar is largely uniform across Texas (i.e., it does not have the same degree of spatial variability that wind does), the chosen locations for solar have negligible variations in mean capacity factor and moderate increases in correlation with residual demand for increasingly strict carbon emissions reductions. These results indicate that at a continental scale, energy system planners can leverage the spatiotemporal variability of both wind and solar to reduce system costs; at regional scale, wind has more spatial spatiotemporal variability than solar, which can be leveraged to reduce system costs.

In the supplemental information, we consider for ERCOT two additional technologies, i.e., nuclear and PGP storage, on top of the original mix of wind, solar, natural gas, and battery storage. We find that the results do not substantially differ after including nuclear and PGP. In fact, based on near-current costs, nuclear and PGP come into play only for deep decarbonization scenarios. The statistical analysis also shows that the inclusion of nuclear and PGP does not change our primary findings. Chosen locations for wind have lower mean capacity factors and higher correlations with residual demand for increasingly strict carbon emissions reductions. For solar at a small geographical scale, the chosen locations have instead negligible variations in mean capacity factor and moderate increases in correlation with residual demand, consistent with the ERCOT results without nuclear and PGP included.

**Figure 3:**
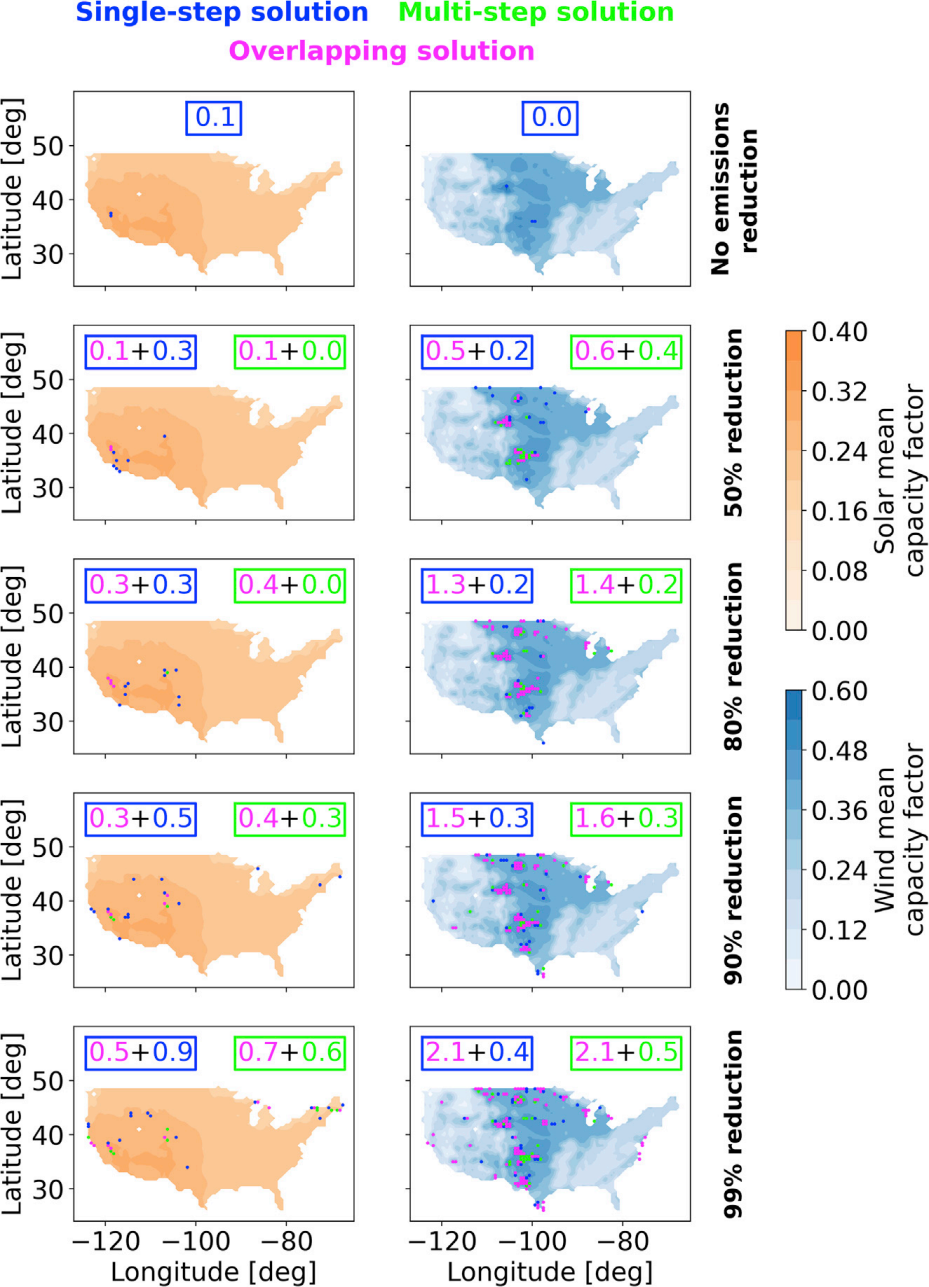
Locations of the wind and solar installations selected by our optimizer for the different emissions reduction cases For each map, we show the spatial distribution of the wind or solar mean capacity factor along with dots indicating the locations where generation capacity was installed in the multi-step and single-step least-cost solution. Overlapping optimal locations from these two approaches are plotted in fuchsia. The optimal, non-overlapping solutions of multi-step and single-step optimizations are plotted in green and blue, respectively. In each panel, we also indicate how much total normalized capacity is installed in the respective colored grid cells for both the multi-step and single-step optimizations.

Lastly, in Figure 3, we show the locations of the wind and solar installations selected by our optimizer for the different emissions reduction cases. For each map, we show the spatial distribution of the wind or solar mean capacity factor along with dots indicating the locations where generation capacity was installed in the least-cost solution. We highlight differences between multi-step and single-step solutions by using different colors for overlapping and non-overlapping solutions. Note that most of the locations for wind installations are shared between the multi-step and single-step optimizations, while the same is not always true for solar, where the selected locations of multi-step and single-step optimizations do not overlap as much.

For the case without emissions limits, the installed capacity of both wind and solar is limited and concentrated in areas with high mean capacity factors, namely, the Central U.S. for wind and the Western U.S. for solar. As emissions reductions are enforced and natural gas generation phases out, more locations that are spread over regions with relatively high mean capacity factors are selected by the optimizer. Most of the locations for wind installations are in the Central and Midwest U.S. with a few others on the West and East coasts. As we have seen in the statistical analysis, wind installations tend to have a larger range in capacity factors and correlations with residual demand for deep decarbonization levels in comparison to solar. This is reflected in wind being sited over regions that do not have the highest capacity factors. For solar, most of the installations are in the Western U.S. with a few others in the Northeast U.S. These lower solar capacity factor locations in the Northeast U.S. show that for systems with lower flexibility, the regions with the highest capacity factors are not always the preferred ones. As the flexibility provided by natural gas diminishes, the correlation between generation and times when other generation is insufficient to meet demand becomes more important. These solar installations in the Northeast U.S. act to broaden the net solar generation profile by adding generation in the Eastern most regions of the U.S. where the sun predictably rises earliest each day. Solar in the East thus helps provide relatively consistent early morning power while the Western U.S. is unlit.

### Limitations of the study

In this study, we consider an idealized electricity system where the sources of energy can be natural gas, wind, and solar, while energy can be stored in batteries or curtailed when the generation exceeds the demand. For this system, we consider free lossless electricity transmission to give the greatest possible advantage to variable wind and solar resources relative to competing technologies. Because we do not include the constraints of grid infrastructure (Millstein et al., 2021), generation and curtailment values are likely to be underestimates. In fact, our simulations are not intended to guide siting decisions for real-world applications, but rather to quantify the importance of long-term planning and the potential advantage of considering the wind and solar resource spatiotemporal variability, complementarity, and correlation with residual demand. Grid infrastructure will be an important consideration in determining which wind and solar sites are eventually developed and connected to the grid. Our analysis shows that long-term planning may benefit from routing transmission not only toward regions with only high mean capacity factors but also toward regions with substantial capacity and good alignment with anticipated future residual load. If done well, this could avoid a degree of undesirable technological lock-in.

Further, electricity delivery timing is only one of many essential grid services that will need to be delivered by wind and solar plants in the future. In addition to balancing supply and demand, future systems with substantial wind, solar, and energy storage must supply frequency and voltage control, ramping, black-start, automatic generation control, and other reliability services in order to manage the entire grid (Aghaei and Alizadeh, 2013; Huber et al., 2014; Kumar et al., 2019; Su and El Gamal, 2013). Here, we conduct only a macro-scale analysis that allows us to assess and disentangle high-level impacts of increasingly strict emissions limits on the optimal siting of distributed wind and solar generation. These impacts are relevant for energy system planners and policymakers as wind and solar photovoltaic are projected to be the dominant generation sources in decarbonized electricity systems.

In this study, we also do not consider offshore wind development or land use constraints such as high population density areas, protected lands (e.g., parks, wilderness), highly productive farmlands, areas with high environmental conservation value, or areas unsuitable for construction (e.g., wetlands, mountain slopes). The deployment of wind on land could change significantly in more restrictive siting scenarios (Lopez et al., 2012; Rinne et al., 2018). Nevertheless, the potential for wind energy far exceeds current U.S. electricity needs even in highly constrained scenarios (Lopez et al., 2021). Offshore wind offers a good opportunity as winds are generally stronger and more persistent over oceans (Pryor et al., 2021).

Lastly, we use technology costs taken from the U.S. Energy Information Administration (U.S. Energy Information Administration (EIA), 2020) and Lazard’s levelized cost of storage report (Lazard, 2019). We use the Energy Information Administration as an objective source for cost estimates; note, however, that a growing body of research is finding that the cost of clean energy is falling faster than has been forecasted by experts (Meng et al., 2021). As these costs decline and the ratio of costs between the different clean technologies changes, the exact quantity of each technology that would be deployed in a least-cost system would change. This is why we do not emphasize the exact quantity of wind or solar in the scenarios and instead emphasize the trends. Our results are intended to be taken in a qualitative manner.

## Discussion

In this paper, we show potential advantages of long-term site planning of wind and solar power plants when relying on them to decarbonize an electricity system. We use a macro-scale energy model to evaluate capacities and dispatch in least-cost electricity systems with distributed wind and solar generation, energy storage, and natural gas generation with increasingly strict carbon emissions limits. This macro-scale analysis allows us to assess and disentangle high-level impacts of increasingly strict emissions limits on the optimal siting of distributed wind and solar generation. These impacts are relevant for energy system planners and policymakers as wind and solar photovoltaic, according to various energy system pathways (Frew et al., 2016; Jacobson et al., 2017; Larson et al., 2020), are projected to be the dominant generation sources in decarbonized electricity systems.

In our simulations with no emissions reduction constraints, natural gas is the dominant energy source, and its use provides a high degree of flexibility to the system so that the few locations with wind and solar installations are characterized by high capacity factors. As generation from natural gas phases out because of stricter emissions limits and electricity systems increasingly rely on wind and solar generation, an increasing fraction of additional wind and solar generation is curtailed, and optimal locations for wind and solar generation have higher correlation with residual demand and lower capacity factors. Multi-step and single-step optimizations for different emissions reduction targets demonstrate that consideration of long-term needs in wind and solar siting decisions results in less mean generation, less mean curtailment, and lower system cost.

This study suggests that when there is a substantial amount of flexible generation (or dispatchable storage) on a grid with widely available high-voltage transmission and negligible losses, the primary geophysical factor governing the value of wind and solar infrastructure at a continental scale is resource amount. With a high degree of flexibility in the system, wind and solar generation can safely be installed in regions with the highest capacity factors without considering correlation with residual demand. Shallow decarbonization (i.e., 50% emissions reduction) results in only a slightly higher system cost and almost negligible curtailment because natural gas provides sufficient grid flexibility. When we get to deep decarbonization, the need to meet demand without dispatchable generation means that the quality of the resource becomes important, and maximizing quantity of generation can yield suboptimal results. With little flexibility in the system, wind and solar assets can have greater value if locations are chosen where power generation correlates with residual demand (i.e., demand minus generation by variable renewables). For smaller geographical regions, optimal locations for wind show mean capacity factors that generally decrease for increasingly strict emissions reductions, while correlation with residual demand increases. Solar, in contrast, is largely uniform and does not have a lot of spatial variability that can be leveraged to reduce the system costs with strategically selected locations.

Real-world siting decisions would require detailed analysis of both current conditions and anticipated future conditions, multi-decadal analyses, and more granular energy system models. In this study, we showed in an idealized setting how siting decisions made with foresight can lead to more efficient asset allocation. Such findings could lead to other important questions that would need to be answered as we move to decarbonized energy systems with a high penetration of variable wind and solar generation. For example, if wind and solar resources in remote locations can help reduce the cost of deeply decarbonized energy systems, it becomes important to understand how that reduction would compete with the cost of adding or expanding transmission in places where ultrahigh-voltage lines are not already available. If ultrahigh-voltage lines were available, it may be that the complementarity of wind and solar resources, enabled on a large scale, could reduce the need for construction of dispatchable generation plants and physical storage such as battery packs. One further aspect that deserves consideration is related to the demand time series. Changes in demand patterns given, for example, by the electrification of the transportation or heating sector could have an influence as big as changes in generation patterns in determining where the next best location is for wind or solar. Lastly, we have performed a least-cost analysis in the absence of socio-political constraints on the construction of electricity generation or transmission infrastructure. In the United States, and in other countries, there are often substantial socio-political challenges in siting such facilities. While our study does not attempt to simulate these challenges, our study highlights the importance of considering, in siting decisions, not only the benefit such infrastructure would provide today, but also where such infrastructure might provide the most benefit over the long term.

Many factors must be considered when choosing sites for expansion of wind and solar generation capacity. Our study suggests that there may be value in including in this list consideration of how residual demand will evolve over time as the flexibility now provided by natural gas is removed from the electricity system. Such consideration will place a higher value on the quality of the wind or solar resource, specifically its correlation with residual demand, and lower value on the mere quantity of wind or sun available. This analysis can be helpful in guiding energy system planners and decision makers for developing least-cost systems that leverage not only wind and solar resource amount (resource quantity) but also their spatiotemporal variability, complementary, and correlation with residual demand (resource quality). For example, system planners may want to increasingly geographically diversify wind and solar farms as energy systems decarbonize. Consideration of the quality wind and solar resources available at each site, and not only the quantity of wind or solar power available, could help buffer against simultaneous wind or solar lulls across a portfolio of wind and solar assets.

## STAR★Methods

### Key resources table

**Table:** 

REAGENT or RESOURCE	SOURCE	IDENTIFIER
**Software and algorithms**		

Macro-scale energy model (MEM)	Ken Caldeira’s research group	https://github.com/carnegie/MEM_public/tree/Antonini_et_al_2022
Gurobi optimizer	Gurobi optimization	https://www.gurobi.com/products/gurobi-optimizer/

**Cost assumptions**		

Wind, solar, and natural gas technologies	Energy Information Administration (EIA)	https://www.eia.gov/outlooks/archive/aeo20/assumptions/pdf/electricity.pdf
Battery technology	Lazard’s levelized cost of storage report	https://www.lazard.com/media/451087/lazards-levelized-cost-of-storage-version-50-vf.pdf

**Input data**		

Reanalysis weather data	NASA’s MERRA-2	https://doi.org/10.1175/JCLI-D-16-0758.1
Electricity demand data	U.S. balancing authorities	https://doi.org/10.1038/s41597-020-0483-x

### Resource availability

#### Lead contact

Further information and requests for resources should be directed to and will be fulfilled by the lead contact, Enrico Antonini (eantonini@carnegiescience.edu).

#### Materials availability

This study did not generate new materials.

### Method details

**Figure 4:**
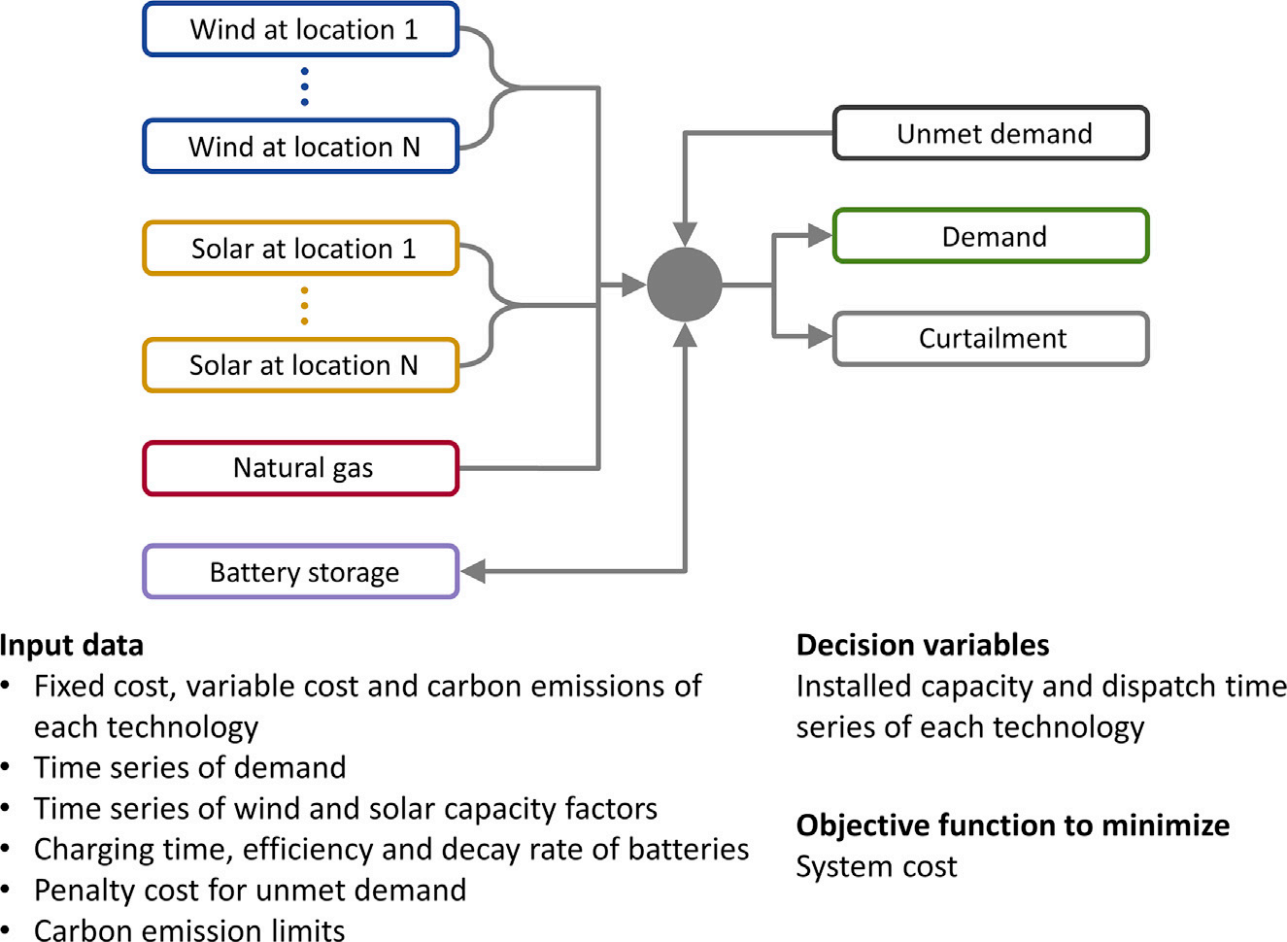
Illustration of the macro-scale energy model used to understand the optimal siting of wind and solar generation while decarbonizing electricity systems In this idealized system, the sources of energy can be natural gas (aggregated), wind, and solar (distributed), while energy can be stored in batteries (aggregated) or curtailed when the generation exceeds the demand (aggregated). The spatial distribution of wind and solar generation is evaluated based on the subdivision of the contiguous U.S. into the grid cells of the MERRA-2 reanalysis dataset, resulting in 2,586 potential locations.

In this study, we use a macro-scale energy model called MEM (Dowling et al., 2020; Henry et al., 2021; Rinaldi et al., 2021; Yuan et al., 2020), illustrated in Figure 4. It includes a set of electricity generation facilities and a firm electricity load in the form of an hourly demand time series. For each generation facility, the model requires cost assumptions. For wind and solar electricity generation facilities, the model also requires hourly capacity factor time series. The model uses a least-cost linear optimizer to find the installed capacities and hourly dispatches for all electricity generation facilities included in the system. Curtailment is allowed for wind and solar electricity generation when supply exceeds demand, resulting in a loss of energy. The model includes an unmet demand component represented with a penalty cost (10 $/kWh). This feature is useful to provide some small degree of flexibility to prevent the situation where extremely costly and infrequent electricity supply shortages strongly influences the optimization results. Further, the macro-scale energy system assumes lossless transmission from generation to load. Real-world transmission losses and regionalization of generation and demand would result in increased discrepancies between supply and demand relative to our idealized scenario. The governing equation of the macro-scale energy model that we use are provided below. Notice that the macro-scale energy model is a closed electricity system, and we did not consider flexibility mechanisms associated with the conversion of electricity into heat, fuel, or other types of energy services. We use the term flexibility here to refer to the degree to which the power system can adjust generation by means of storage (e.g., batteries) or dispatchable technologies (e.g., natural gas) in reaction to variability of demand or non-dispatchable technologies (e.g., wind and solar).

We use this model to study a transition to deeply decarbonized electricity systems that rely mostly on wind and solar generation facilities. We are interested in understanding what drives the optimal, least-cost siting of wind and solar installations as natural gas generation phases out because of increasingly strict carbon emissions limits. We consider an idealized electricity system where the sources of energy can be natural gas, wind and solar, while energy can be stored in batteries or curtailed when the generation exceeds the demand. Our focus is on resource quality and not on future geographic relationships among resource quality, transmission infrastructure, and demand. Therefore, we consider a system with free lossless electricity transmission. While this assumption may not be currently realistic for some countries or continents, such as the US, projects for developing continental, ultra-high-voltage power lines that would minimize transmission losses are underway, for example, in China (Americans for a Clean Energy Grid (ACEG), 2020; Bloomberg News, 2020) or are being proposed in the U.S. for the coming years (White House, 2021). Free lossless transmission is instead a better approximation for a smaller region like Texas, which we analyze in the supplemental information. In such systems with lossless transmission, location matters primarily for wind and solar power, while batteries, natural gas, and demand can be considered in aggregate because their contribution to the system cost is not affected by their spatial variability. Cost assumptions for our analysis are reported in Table 1.

**Table 1:** Economic, cost, and carbon assumptions

	Natural gas	Wind	Solar	Battery
Capacity (fixed) cost type	Power capacity [$/kW]	Power capacity [$/kW]	Power capacity [$/kW]	Energy capacity [$/kWh]
Capacity (fixed) cost	954	1,319	1,331	366
Project life [yrs]	30	25	25	10
Discount rate [%]	7	7	7	7
Capital recovery factor [%]	8.1	8.6	8.6	14.2
Fixed O&M cost [$/kW-yr]	12.1	26.2	15.2	12.3
Variable O&M cost [$/kWh]	0.00186			
Fuel cost [$/kWh]	0.0191			
Round-trip efficiency				0.9
Self-discharge rate [% per hour]				0.00001
Charging time [h]				4
**Carbon emissions [kgCO**_**2**_**/kWh]**	**0.461**			
**Fixed cost [$/kW/h]**	**0.0102**	**0.0159**	**0.0148**	**0.00735**
**Variable cost [$/kWh/h]**	**0.021**			

Values taken from the U.S. Energy Information Administration (U.S. Energy Information Administration (EIA), 2020) and Lazard’s levelized cost of storage report (Lazard, 2019).

The spatial distribution of wind and solar generation is evaluated by subdividing the contiguous U.S. into the grid cells of the MERRA-2 reanalysis dataset (Gelaro et al., 2017). The dataset has a spatial resolution of 0.5° latitude x 0.625° longitude. Over the contiguous U.S., there are 2,586 grid cells with horizontal dimensions ranging from about 55 km × 45 km to 55 km × 62 km. We refer to each of these grid cells as a “location”. For each location, we calculate hourly capacity factor time series of wind and solar. For each location, we also set maximum capacity limits to prevent unrealistic, concentrated installations of wind or solar generation from being selected by the optimizer. These limits are given by geophysical constraints on the available mean wind and solar power per unit land and depend on the local characteristics of the solar irradiance and wind resource. While complex and sophisticated estimations of these limits are available that take into consideration many factors in addition to resource quality (Antonini and Caldeira, 2021a, 2021b; Mackay, 2013; Miller et al., 2015), in keeping with the illustrative quality of our analysis, we consider resource quality only; we use two fixed values that are consistent with observations, namely, 1 and 5 W/m^2^ for wind and solar generation, respectively (Miller and Keith, 2018). The maximum capacity for each location is calculated by multiplying the maximum power density by the cell area. Note that we do not consider restricted areas with potentially conflicting land uses, such as high population density areas, protected lands (e.g., parks, wilderness), highly productive farmlands, areas with high environmental conservation value, or areas unsuitable for construction (e.g., wetlands, mountain slopes). In general, the resolution of the MERRA-2 dataset does not provide a sufficient level of granularity to consider such constraints. Each grid cell has dimensions that allow some flexibility with regard to the exact location of potential wind and solar power plants while meeting the cell’s total generation.

The capacity factors of wind and solar at each location are calculated as follows. For wind, we assume a wind turbine hub height of 100 m. We estimate the wind speed at the corresponding hub height using a power law and interpolating 10 m and 50 m values retrieved from the MERRA-2 dataset to 100 m. The calculation of wind capacity factor uses a piecewise function consisting of four parts (Clack et al., 2016; Shaner et al., 2018): (i) below the cut-in speed (*u*_*ci*_) of 3 m/s, the capacity factor is zero, (ii) between the cut-in speed of 3 m/s and rated speed (*u*_*r*_) of 12 m/s, the capacity factor is $u^{3}/u_{r}^{3}$, (iii) between the rated speed of 12 m/s and the cut-out speed (*u*_*co*_) of 25 m/s, the capacity factor is 1, and (iv) above the cut-out speed of 25 m/s, the capacity factor is zero. For solar, we calculate the solar zenith angle based on the geographic location and local time. The solar incidence angle is then estimated by considering a single axis tracking solar panel system (Braun and Mitchell, 1983), with a tilt angle of 0° (i.e., horizontal axis of rotation) and a maximum rotation angle of 45° with respect to the vertical direction. The total received solar radiation is composed of direct and diffuse radiation components (McCormick and Suehrcke, 1991), which are calculated based on the solar incidence angle and the incoming solar radiation at both surface and top-of-atmosphere. The panel efficiency then results from the in-panel solar irradiance and the ambient temperature (Huld et al., 2010; Pfenninger and Staffell, 2016).

We consider optimizations over one calendar year. We have hourly time-averaged wind and solar resource data from the year 2019, obtained from the MERRA-2 reanalysis weather dataset (Gelaro et al., 2017) and electricity demand data from the year 2019, obtained from balancing authorities across the contiguous U.S. (Ruggles et al., 2020). We show results for 2019 in the main body of the text, and show results for 2016, 2017, and 2018 in the supplemental information to demonstrate that results are consistent despite the expected inter-annual variability of wind and solar resources (Ruggles and Caldeira, 2022). While a multi-decadal analysis with projections of future demand and resources would provide more realistic results, our idealized approach can reveal and disentangle system-level relationships and characteristics of optimal siting of distributed wind and solar generation in a decarbonized electricity system.

The decarbonization of electricity systems in our model is implemented with increasingly strict carbon emissions limits. The generation from natural gas entails a certain amount of CO_2_ for each kWh produced. We first calculate the emissions that would be released by supplying all the electricity with natural gas generation, and we then set reductions of 50, 80, 90, and 99% of the original emissions. The optimization to meet those emissions constraints is run in two different ways. The first one considers a multi-step optimization, i.e., as new, stricter emissions limits are imposed, the built infrastructure from the less strict constraint is kept, and new wind and solar generation is added to replace the missing generation from natural gas. The second approach considers instead a single-step optimization, i.e., we determine the system infrastructure for a given emissions constraint that would be in place if we could build such a system from the beginning. The comparison between these two approaches will allow us to assess the impact of a short vs. long term system planning strategy.

#### Macro-scale energy model (MEM)

In this study, we use a macro-scale energy model. It includes a set of electricity generation facilities and a firm electricity load in the form of an hourly demand time series. For each generation facility, the model requires cost assumptions, carbon emissions assumptions, and hourly capacity factor time series. The model uses a linear optimizer to find the installed capacities and hourly dispatches, for all electricity generation facilities included in the system, that minimize total system cost. Curtailment is allowed for variable renewable energy generation when supply exceeds demand. The model includes an unmet demand component represented with a penalty cost (10 $/kWh).

Each technology (natural gas, *n*, wind, *w*, solar, *s*, or battery, *b*) is characterized by a fixed hourly cost resulting from the capital expenditure, *c*_*capital*_, and operation and maintenance costs, *c*_*O&M*_:

(Equation 1) $$\begin{array}{l} c_{\mathrm{fixed}}^{n,w,s,b}=\frac{\gamma c_{\mathrm{capital}}^{n,w,s,b}+c_{O\&M}^{n,w,s,b}}{h}\text{,} \end{array}$$

where *h* is the number of hours per year and *γ* is the capital recovery factor, defined as:

(Equation 2) $$\begin{array}{l} \gamma =\frac{i{\left(1+i\right)}^{n}}{{\left(1+i\right)}^{n}-1}\text{,} \end{array}$$

where *i* is the discount rate and *n* is the asset lifetime in years. Natural gas is also characterized by a variable cost, *c*_*variable*_.

In the model, we introduce constraints on the installed capacity for both wind and solar, *C*:

(Equation 3) $$\begin{array}{l} 0\;\leq \;C^{w,s}\;\leq \;C_{max}^{w,s} \end{array}$$

as well as constraints on the dispatch time series for natural gas, wind and solar at each time step, *D*_*t*_:

(Equation 4) $$\begin{array}{l} 0\;\leq \;D_{t}^{n,w,s}\;\leq \;C^{n,w,s}\text{.} \end{array}$$

Batteries are characterized by constraints on the energy flowing into them, $D_{t}^{to-b}$:

(Equation 5) $$\begin{array}{l} 0\;\leq \;D_{t}^{to-b}\;\leq \;\frac{C^{b}}{\tau^{b}}\text{,} \end{array}$$

and energy flowing from them, $D_{t}^{from-b}$:

(Equation 6) $$\begin{array}{l} 0\;\leq \;D_{t}^{from-b}\;\leq \;\frac{C^{b}}{\tau^{b}}\text{,} \end{array}$$

where *τ*^*b*^ is the storage charging duration. The total energy available in the batteries, *S*_*t*_, is also constrained by the total capacity:

(Equation 7) $$\begin{array}{l} 0\;\leq \;S_{t}\;\leq \;C^{b}\text{,} \end{array}$$

and the energy flowing from the batteries is affected by the battery decay rate (fraction of energy loss per hour), *δ*^*b*^

(Equation 8) $$\begin{array}{l} 0\;\leq \;D_{t}^{from-b_{t}}\;\leq \;S_{t-1}\left(1-\delta^{b}\right)\text{.} \end{array}$$

The battery storage energy balance is modeled with the following equations, where for *t* = 1:

(Equation 9) $$\begin{array}{l} S_{1}=\left(1-\delta^{b}\right)S_{T}\Delta t+\eta^{s}D_{T}^{to-b}\Delta t-D_{T}^{from-b}\Delta t\text{,} \end{array}$$

and for *t* > 1:

(Equation 10) $$\begin{array}{l} S_{t+1}=\left(1-\delta^{b}\right)S_{t}\Delta t+\eta^{s}D_{t}^{to-b}\Delta t-D_{t}^{from-b}\Delta t\text{,} \end{array}$$

where *η*^*s*^ is the battery electrolysis efficiency.

The whole system energy balance is defined as follows:

(Equation 11) $$\begin{array}{l} \sum_{n,\;w,s}D_{t}^{n,w,s}\Delta t+D_{t}^{from-b}\Delta t+D_{t}^{ud}\Delta t=M_{t}+D_{t}^{to-b}\Delta t+C_{u}, \end{array}$$

where *M*_*t*_ is the demand at time *t*, *C*_*u*_ the curtailment, and $D_{t}^{ud}$ the unmet demand.

Lastly, the objective function to minimize is the system cost:

(Equation 12) $$\sum_{w,s,b}c_{\mathrm{fixed}}^{n,w,s,b}C^{w,s,b}+\frac{\sum_{t}c_{\mathrm{variable}}^{n}D_{t}^{n}}{T}+\frac{\sum_{t}c^{ud}D_{t}^{ud}}{T}$$

where *c*^*ud*^ is the cost for the unmet demand (10 $/kWh).

## Data Availability

• Input data (wind and solar resources and electricity demand) required to reproduce the results reported in this paper were retrieved from the MERRA-2 reanalysis weather dataset (Gelaro et al., 2017) and from balancing authorities across the contiguous U.S. (Ruggles et al., 2020). • Code and instructions required to reproduce the results reported in this paper are available in the GitHub repositories at https://github.com/eantonini/Distributed_wind_and_solar_generation and https://github.com/carnegie/MEM_public/tree/Antonini_et_al_2022. • Any additional information required to reanalyze the data reported in this paper is available from the lead contact upon request.

## Footnotes

Supplemental information can be found online at https://doi.org/10.1016/j.isci.2022.104140.
