# Supplementary material for: The quantity-quality transition in the value of expanding wind and solar power generation
Source: iScience. 2022 Mar 22;25(4):104140. doi: 10.1016/j.isci.2022.104140 (PMC9010648; doi:10.1016/j.isci.2022.104140)
Supplement: Document S1. Figures S1–S26, Tables S1 and S2 [file mmc1.pdf]

iScience, Volume *25*

Supplemental information

The quantity-quality transition in the value of expanding wind and solar power generation

Enrico G.A. Antonini, Tyler H. Ruggles, David J. Farnham, and Ken Caldeira

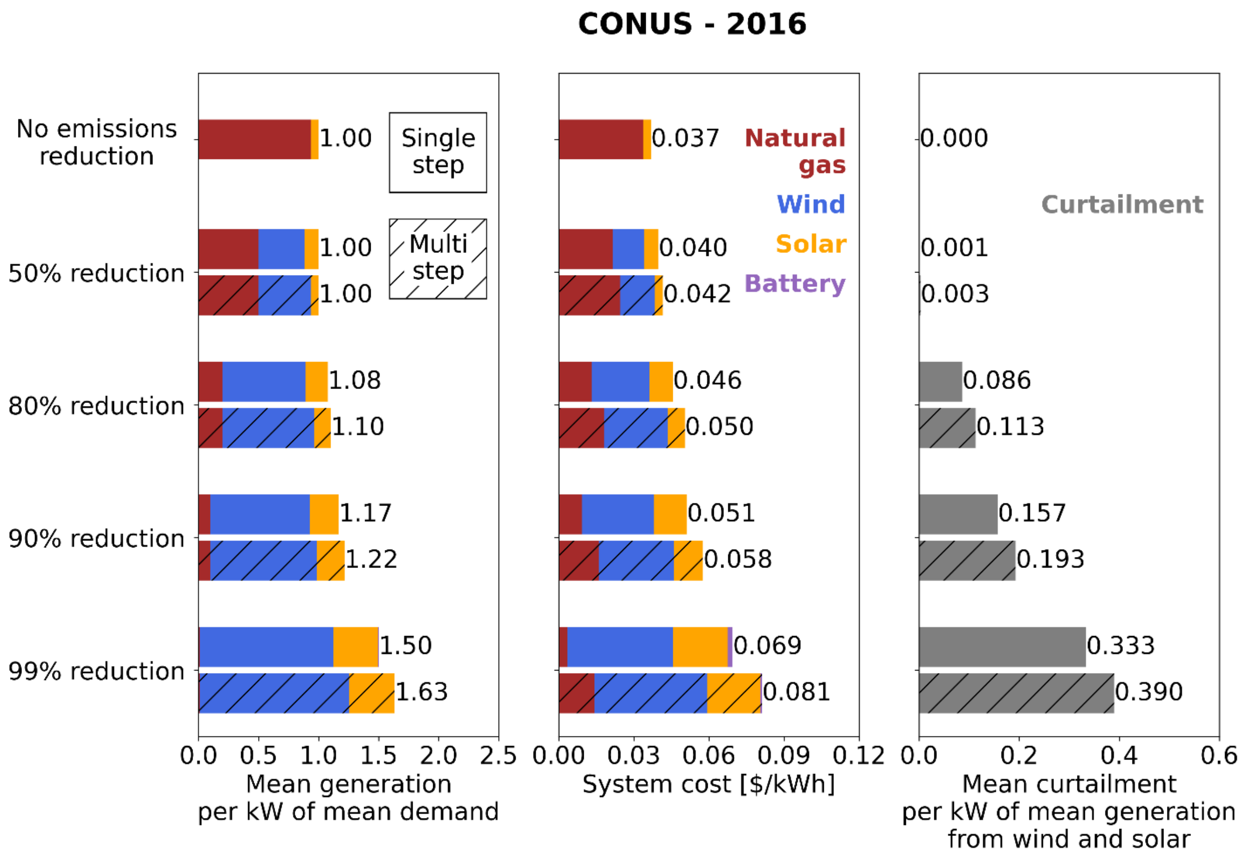

Figure S1. Mean generation, system level cost, and mean curtailment for increasingly strict carbon emissions limits resulting from both multi-step and single-step optimizations for CONUS in the year 2016 when considering wind, solar, natural gas, and battery technologies. This figure is related to Fig. 1. The mean electricity demand is approximately equal to 460 GW.

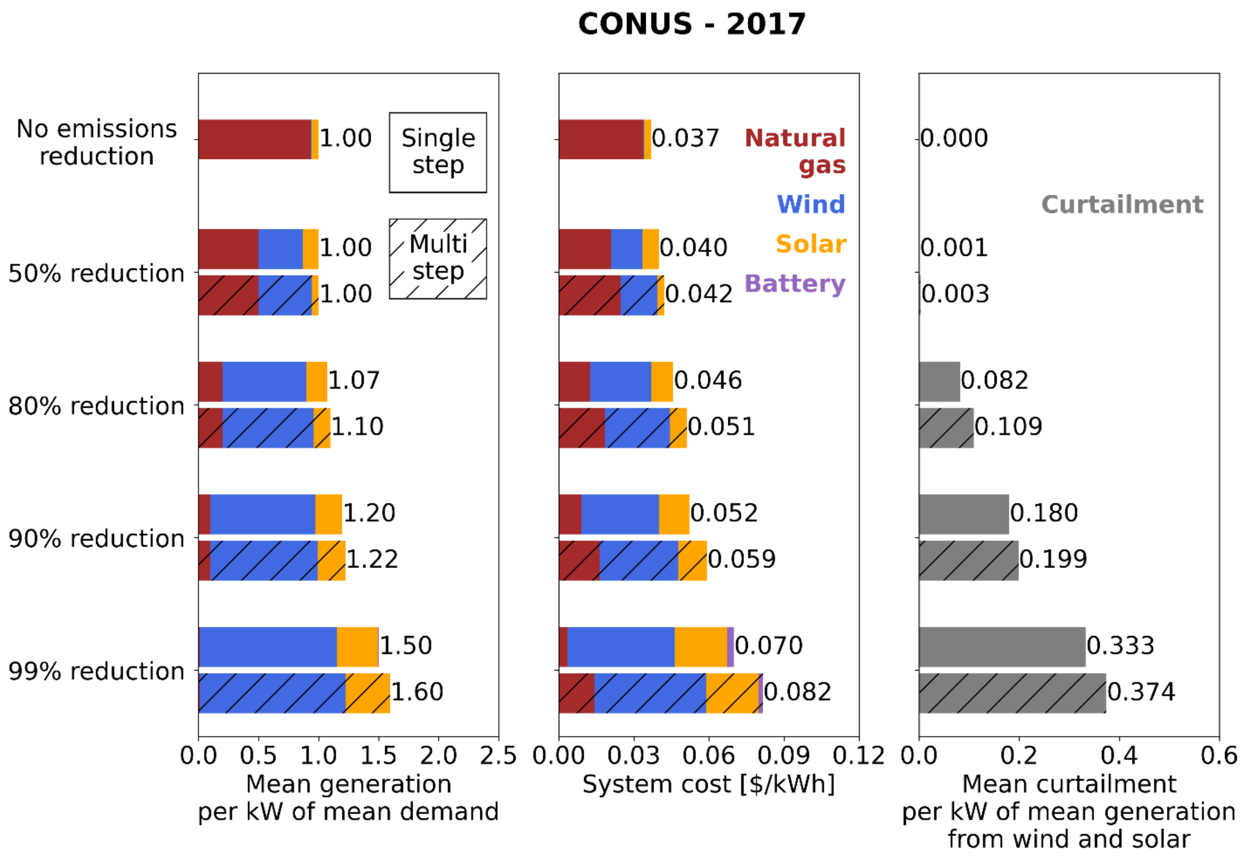

Figure S2. Mean generation, system level cost, and mean curtailment for increasingly strict carbon emissions limits resulting from both multi-step and single-step optimizations for CONUS in the year 2017 when considering wind, solar, natural gas, and battery technologies. This figure is related to Fig. 1. The mean electricity demand is approximately equal to 460 GW.

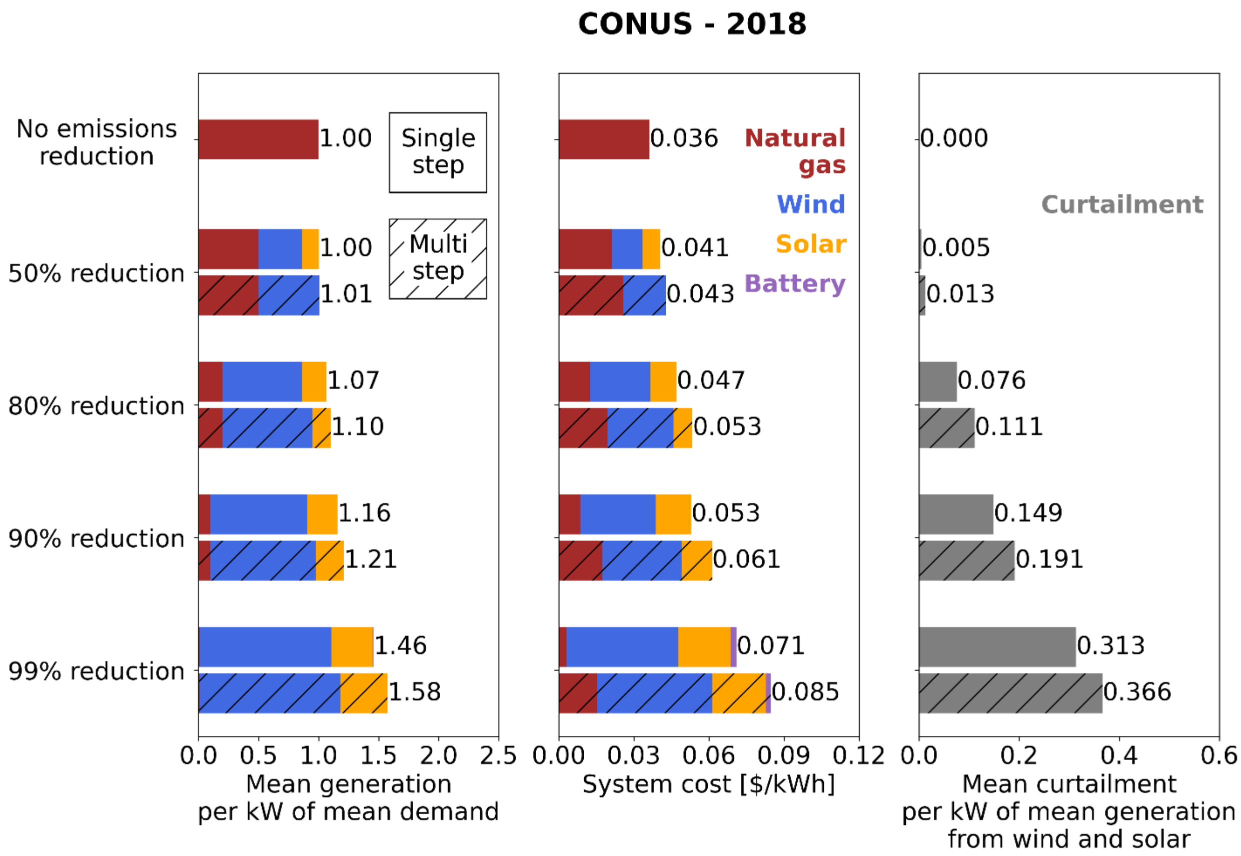

Figure S3. Mean generation, system level cost, and mean curtailment for increasingly strict carbon emissions limits resulting from both multi-step and single-step optimizations for CONUS in the year 2018 when considering wind, solar, natural gas, and battery technologies. This figure is related to Fig. 1. The mean electricity demand is approximately equal to 460 GW.

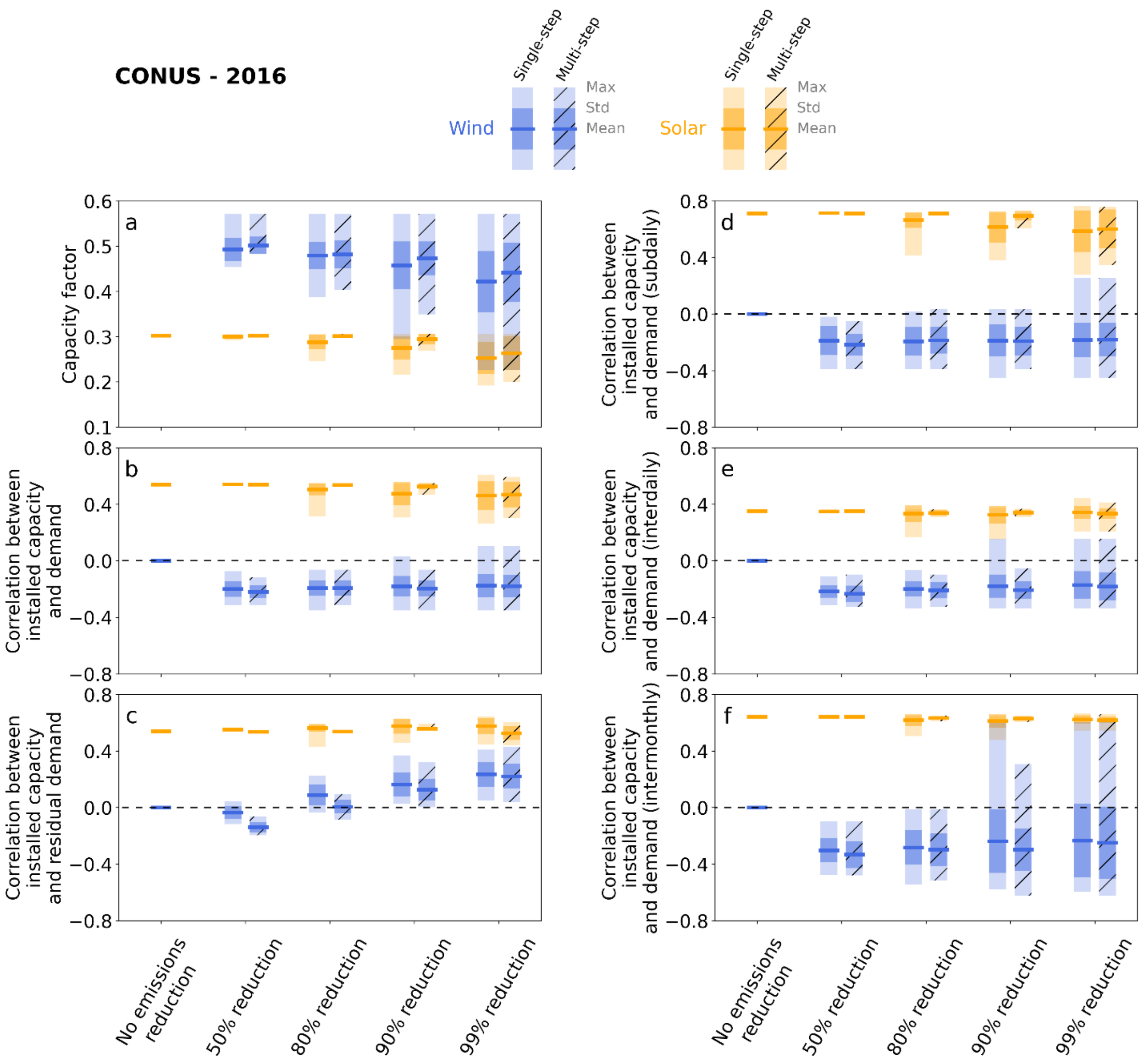

Figure S4. Statistical analysis of the wind and solar capacity factors of the optimized locations for various emission reduction targets for CONUS in the year 2016 when considering wind, solar, natural gas, and battery technologies. This figure is related to Fig. 2. Panel a shows the mean and standard deviation of capacity factors of the chosen locations. Panel b shows the mean and standard deviation of correlation of wind and solar capacity factor time series with the demand time series. Panel c shows the mean and standard deviation of correlation of wind and solar capacity factor time series with the residual demand time series. Panels d, e, and f show the mean and standard deviation of correlations between wind and solar capacity factor time series and the demand time series after a filter has been applied.

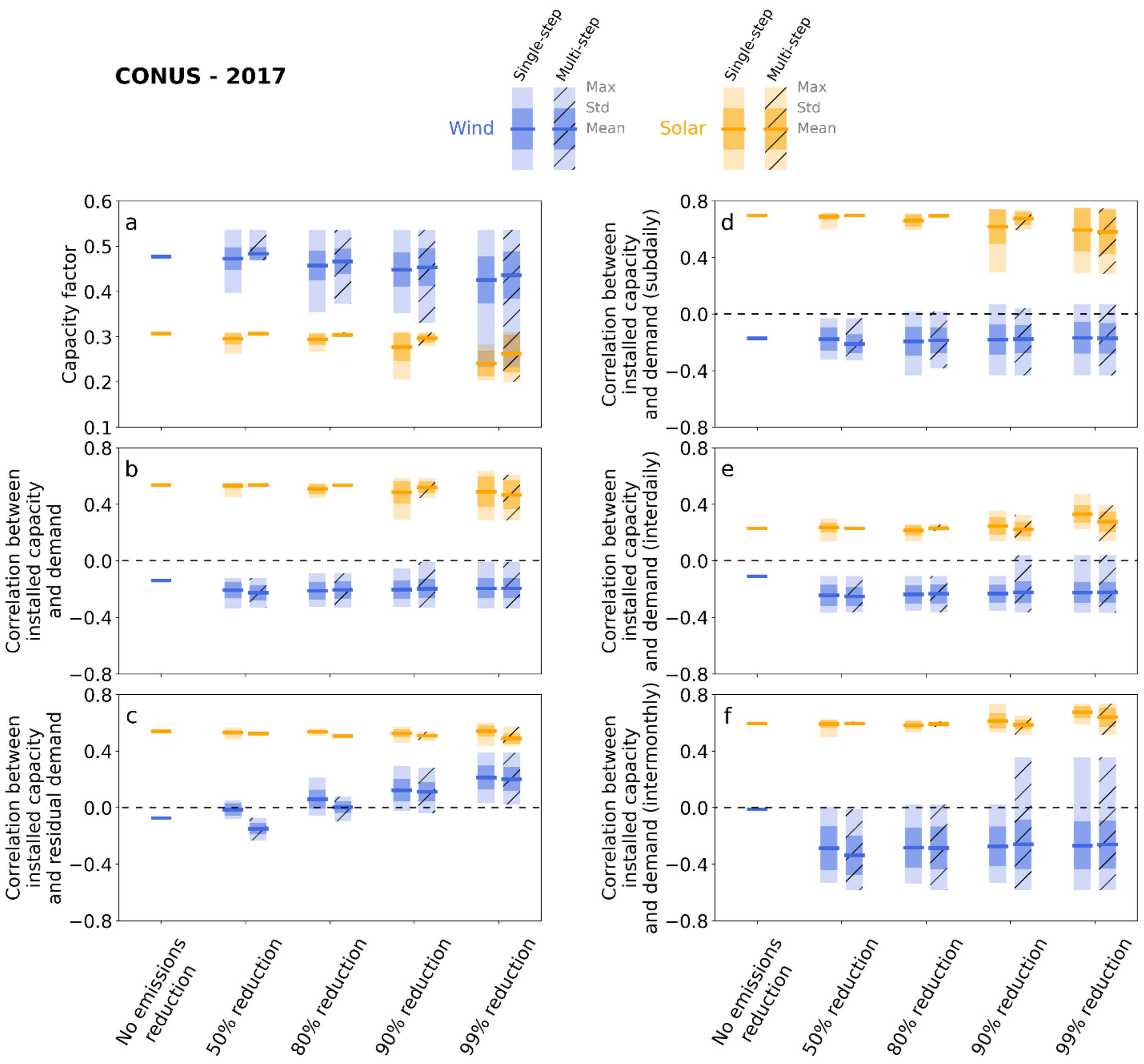

Figure S5. Statistical analysis of the wind and solar capacity factors of the optimized locations for various emission reduction targets for CONUS in the year 2017 when considering wind, solar, natural gas, and battery technologies. This figure is related to Fig. 2. Panel a shows the mean and standard deviation of capacity factors of the chosen locations. Panel b shows the mean and standard deviation of correlation of wind and solar capacity factor time series with the demand time series. Panel c shows the mean and standard deviation of correlation of wind and solar capacity factor time series with the residual demand time series. Panels d, e, and f show the mean and standard deviation of correlations between wind and solar capacity factor time series and the demand time series after a filter has been applied.

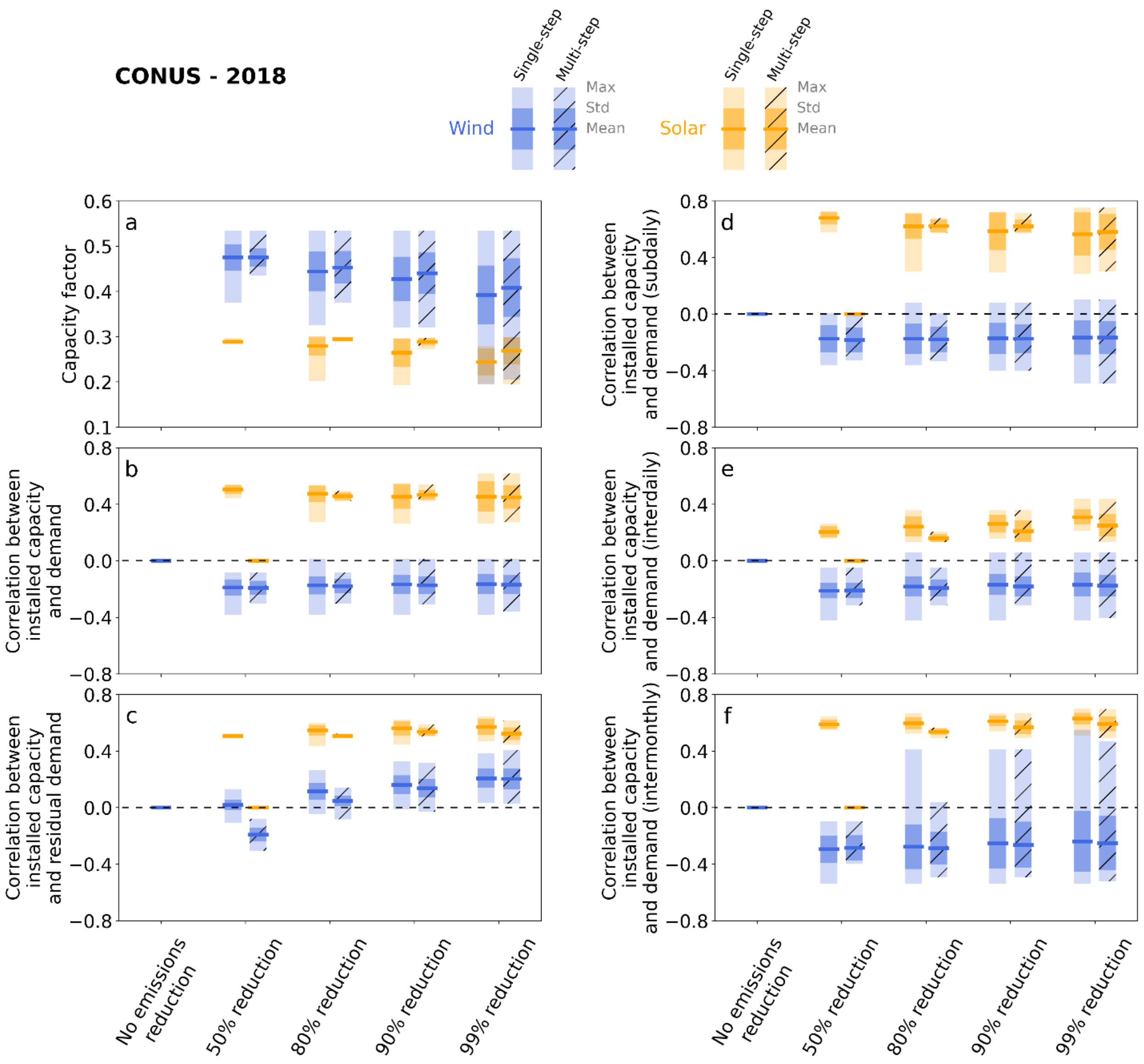

Figure S6. Statistical analysis of the wind and solar capacity factors of the optimized locations for various emission reduction targets for CONUS in the year 2018 when considering wind, solar, natural gas, and battery technologies. This figure is related to Fig. 2. Panel a shows the mean and standard deviation of capacity factors of the chosen locations. Panel b shows the mean and standard deviation of correlation of wind and solar capacity factor time series with the demand time series. Panel c shows the mean and standard deviation of correlation of wind and solar capacity factor time series with the residual demand time series. Panels d, e, and f show the mean and standard deviation of correlations between wind and solar capacity factor time series and the demand time series after a filter has been applied.

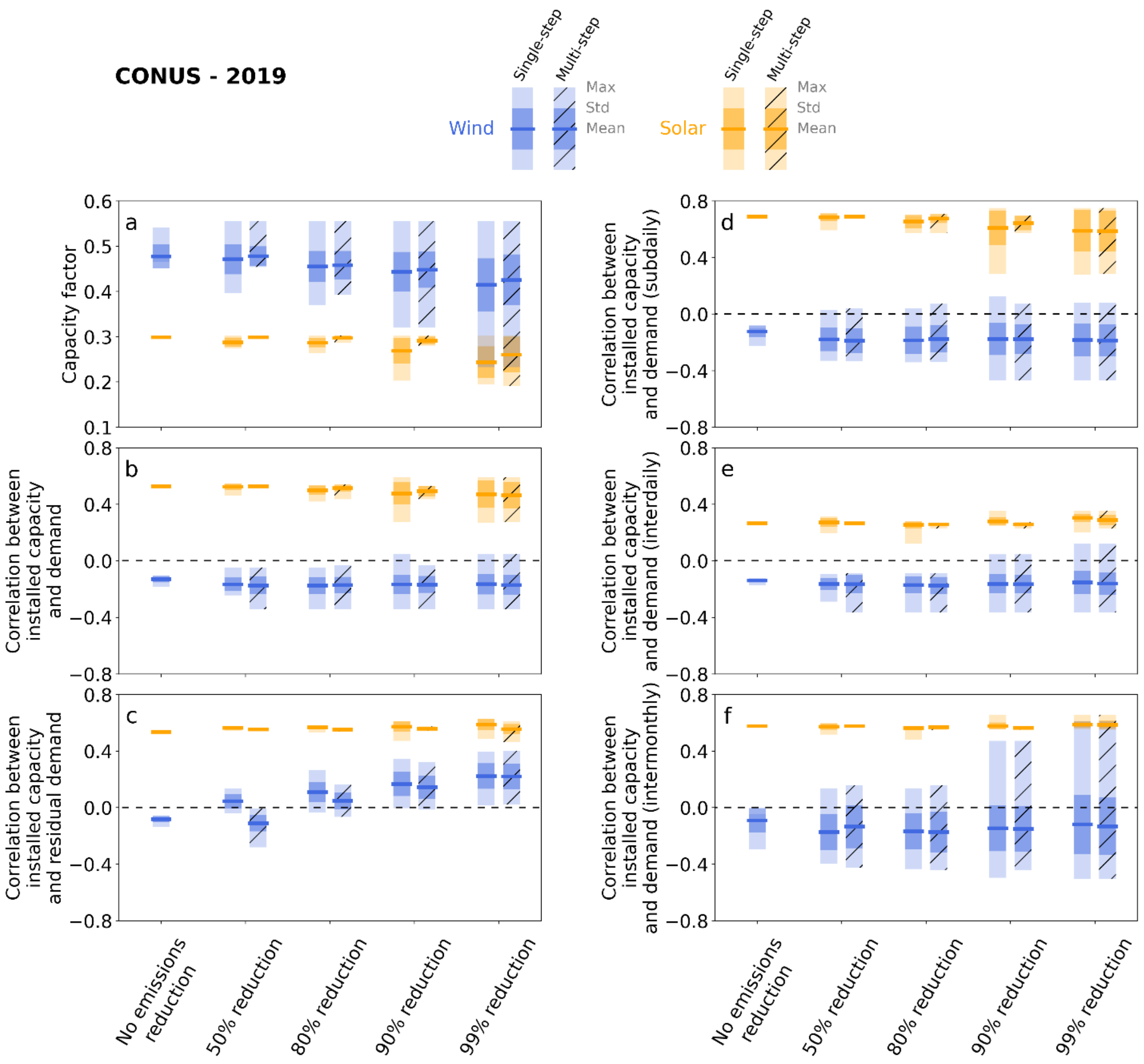

Figure S7. Statistical analysis of the wind and solar capacity factors of the optimized locations for various emission reduction targets for CONUS in the year 2019 when considering wind, solar, natural gas, and battery technologies. This figure is related to Fig. 2. Panel a shows the mean and standard deviation of capacity factors of the chosen locations. Panel b shows the mean and standard deviation of correlation of wind and solar capacity factor time series with the demand time series. Panel c shows the mean and standard deviation of correlation of wind and solar capacity factor time series with the residual demand time series. Panels d, e, and f show the mean and standard deviation of correlations between wind and solar capacity factor time series and the demand time series after a filter has been applied.

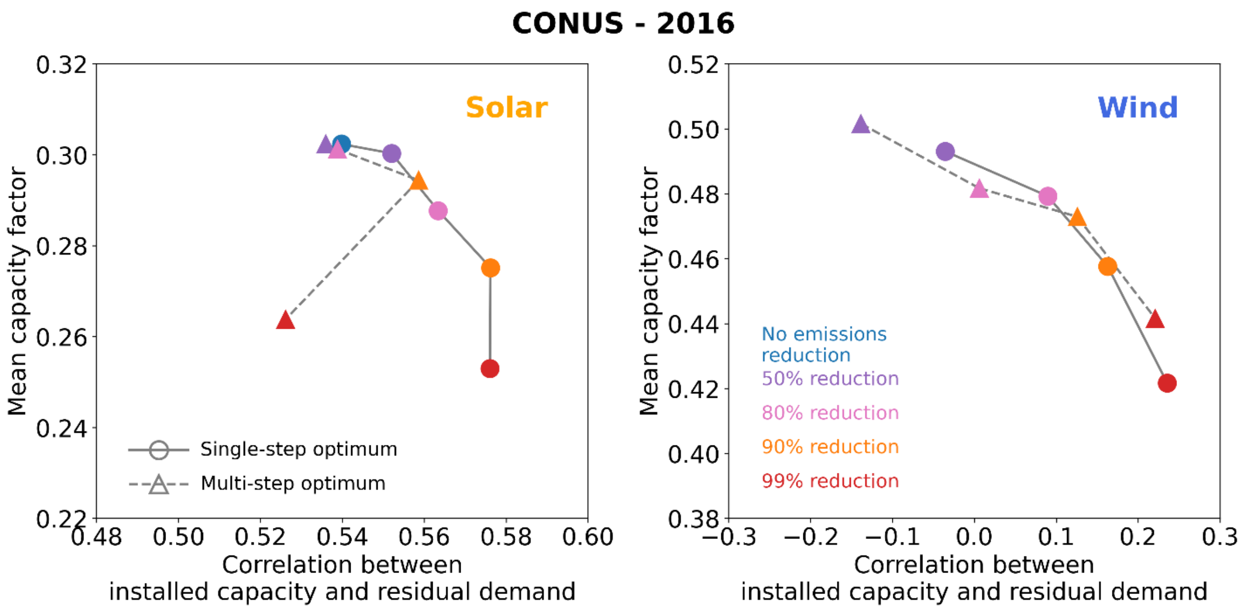

Figure S8. Mean capacity factors of wind and solar installations versus the correlation between the capacity factor time series and the residual demand for different emissions reduction targets for CONUS in the year 2016 when considering wind, solar, natural gas, and battery technologies. This figure is related to Fig. 2. The left panel shows results for the solar installations, while the right panel for wind. For increasingly strict carbon emissions limits, mean capacity factors generally decrease and correlations with residual demand increase. If no marker is shown for a given emissions reduction target, it means that there is no built capacity, or the solution fully coincides with the stricter emissions reduction target (markers overlap).

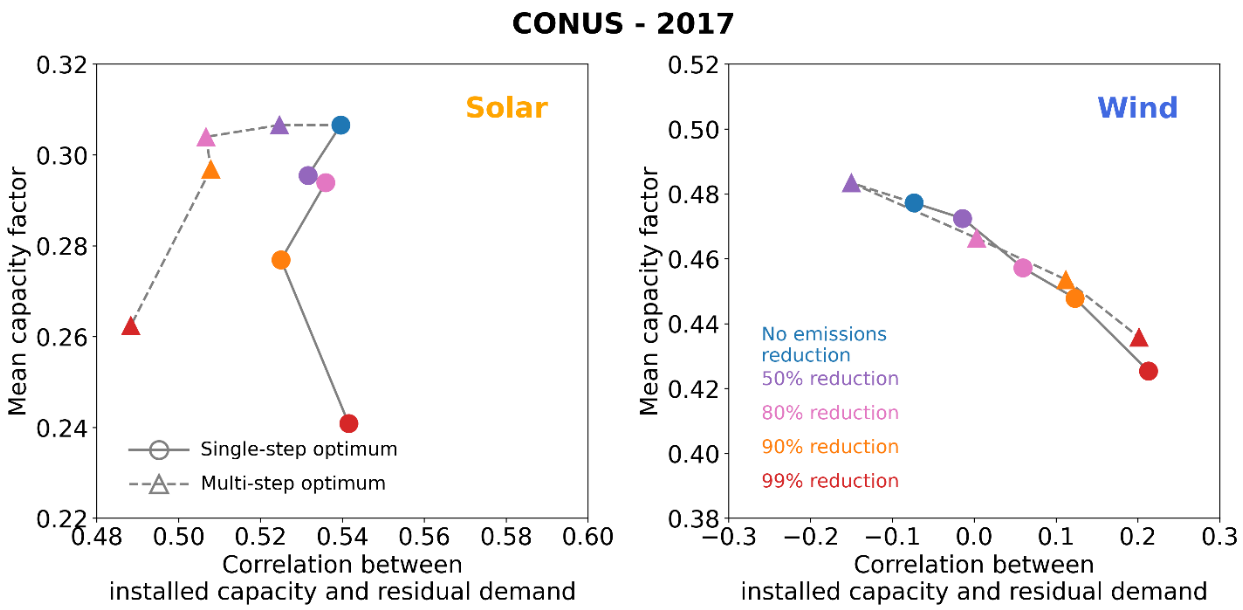

Figure S9. Mean capacity factors of wind and solar installations versus the correlation between the capacity factor time series and the residual demand for different emissions reduction targets for CONUS in the year 2017 when considering wind, solar, natural gas, and battery technologies. This figure is related to Fig. 2. The left panel shows results for the solar installations, while the right panel for wind. For increasingly strict carbon emissions limits, mean capacity factors generally decrease and correlations with residual demand increase. If no marker is shown for a given emissions reduction target, it means that there is no built capacity, or the solution fully coincides with the stricter emissions reduction target (markers overlap).

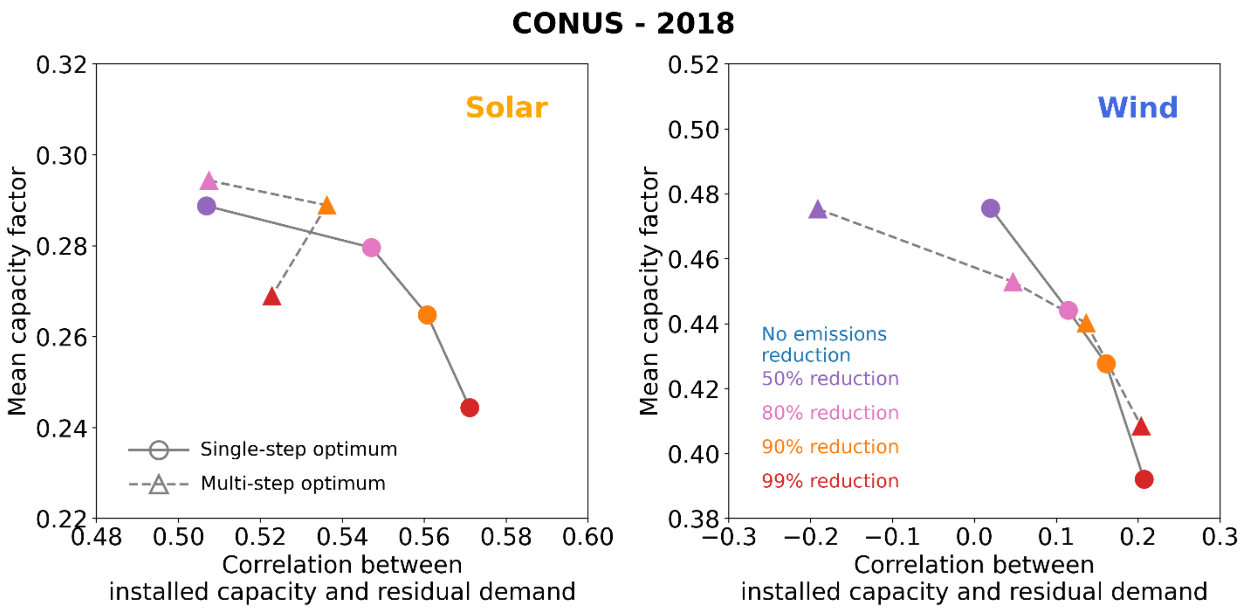

Figure S10. Mean capacity factors of wind and solar installations versus the correlation between the capacity factor time series and the residual demand for different emissions reduction targets for CONUS in the year 2018 when considering wind, solar, natural gas, and battery technologies. This figure is related to Fig. 2. The left panel shows results for the solar installations, while the right panel for wind. For increasingly strict carbon emissions limits, mean capacity factors generally decrease and correlations with residual demand increase. If no marker is shown for a given emissions reduction target, it means that there is no built capacity, or the solution fully coincides with the stricter emissions reduction target (markers overlap).

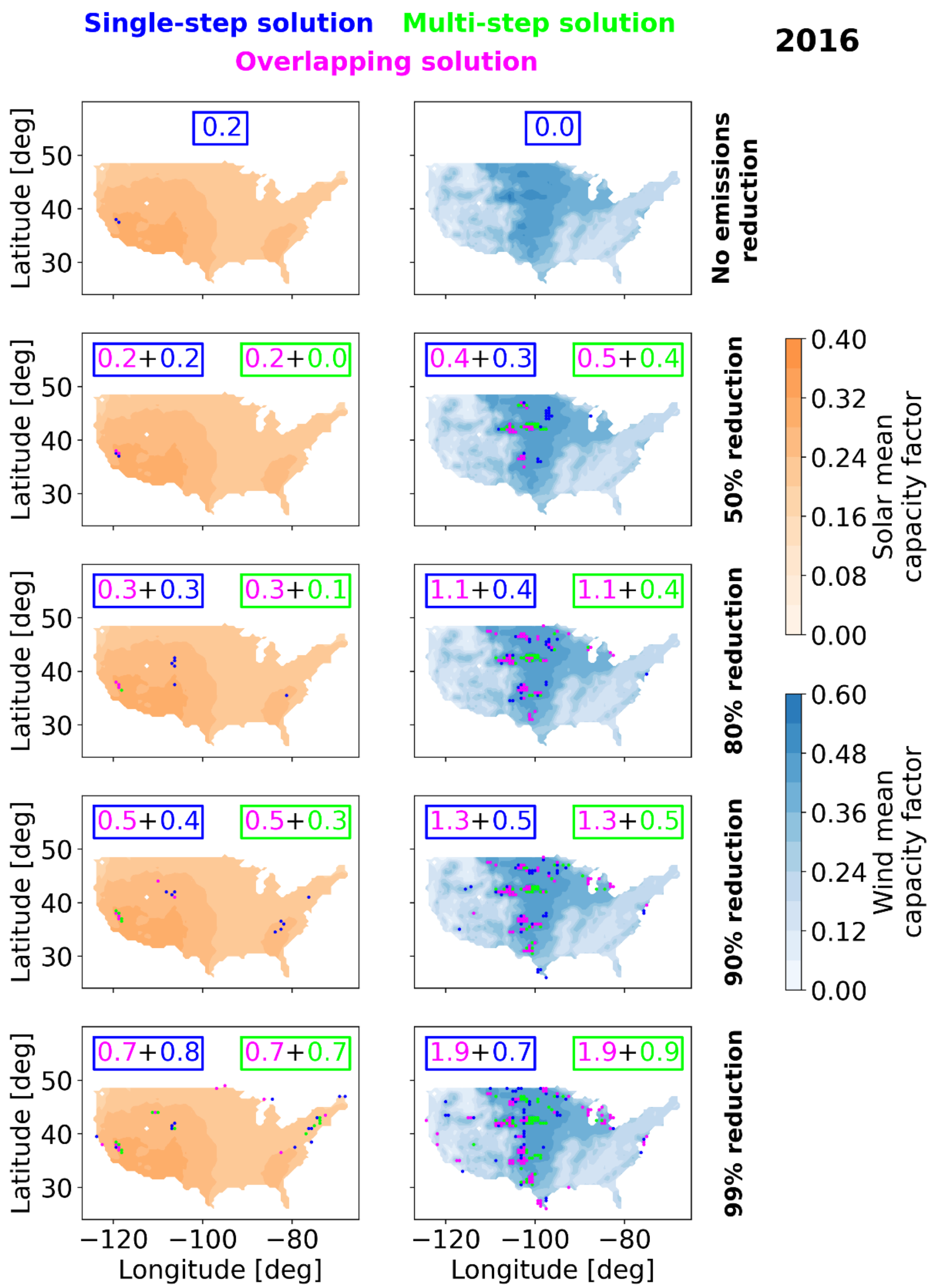

Figure S11. Locations of the wind and solar installations selected by our optimizer for the different emissions reduction cases for CONUS in the year 2016 when considering wind, solar, natural gas, and battery technologies. This figure is related to Fig. 3. For each map, we show the spatial distribution of the wind or solar mean capacity factor along with dots indicating the locations where generation capacity was installed in the multi-step and single-step least-cost solutions.

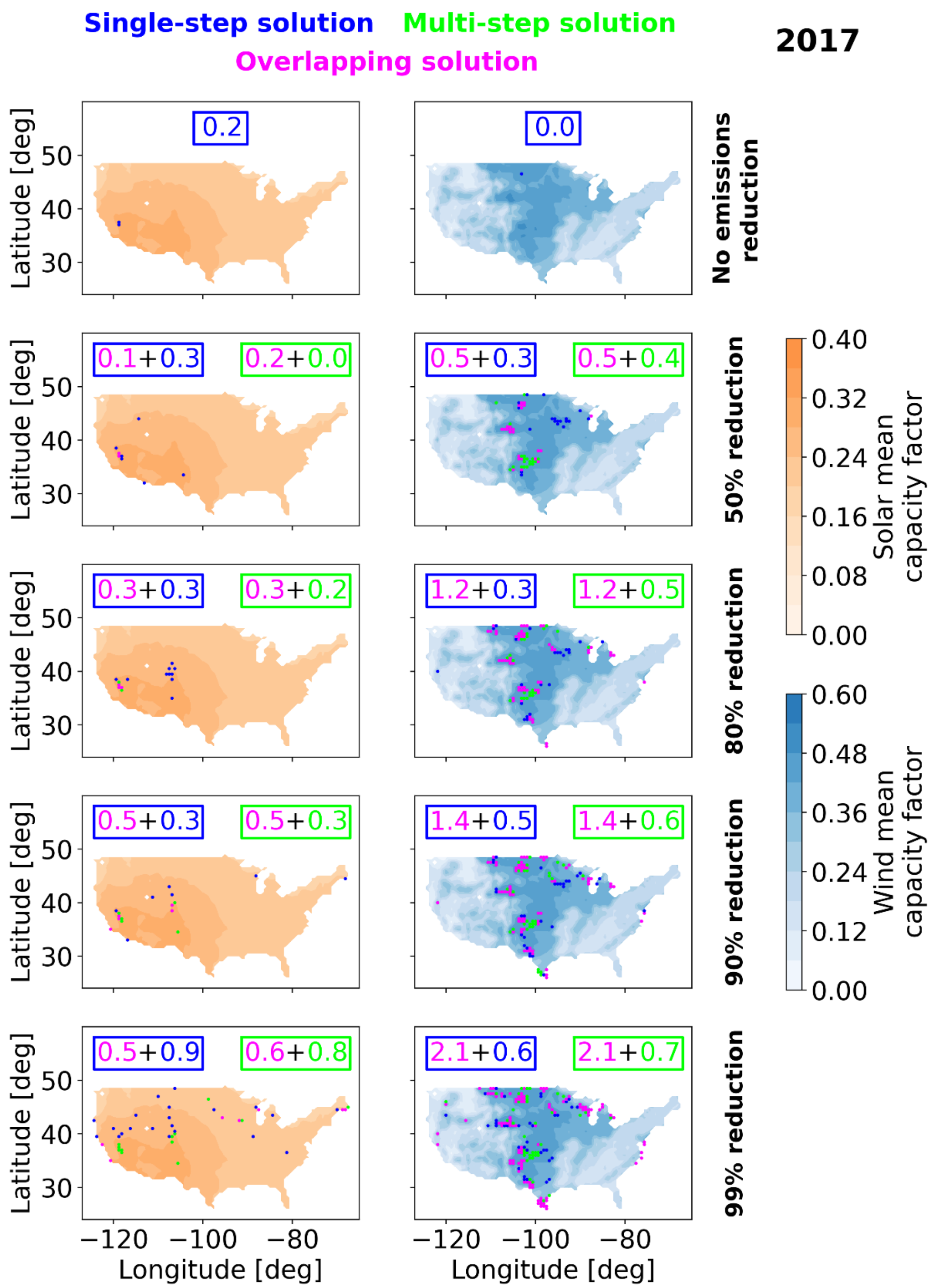

Figure S12. Locations of the wind and solar installations selected by our optimizer for the different emissions reduction cases for CONUS in the year 2017 when considering wind, solar, natural gas, and battery technologies. This figure is related to Fig. 3. For each map, we show the spatial distribution of the wind or solar mean capacity factor along with dots indicating the locations where generation capacity was installed in the multi-step and single-step least-cost solutions.

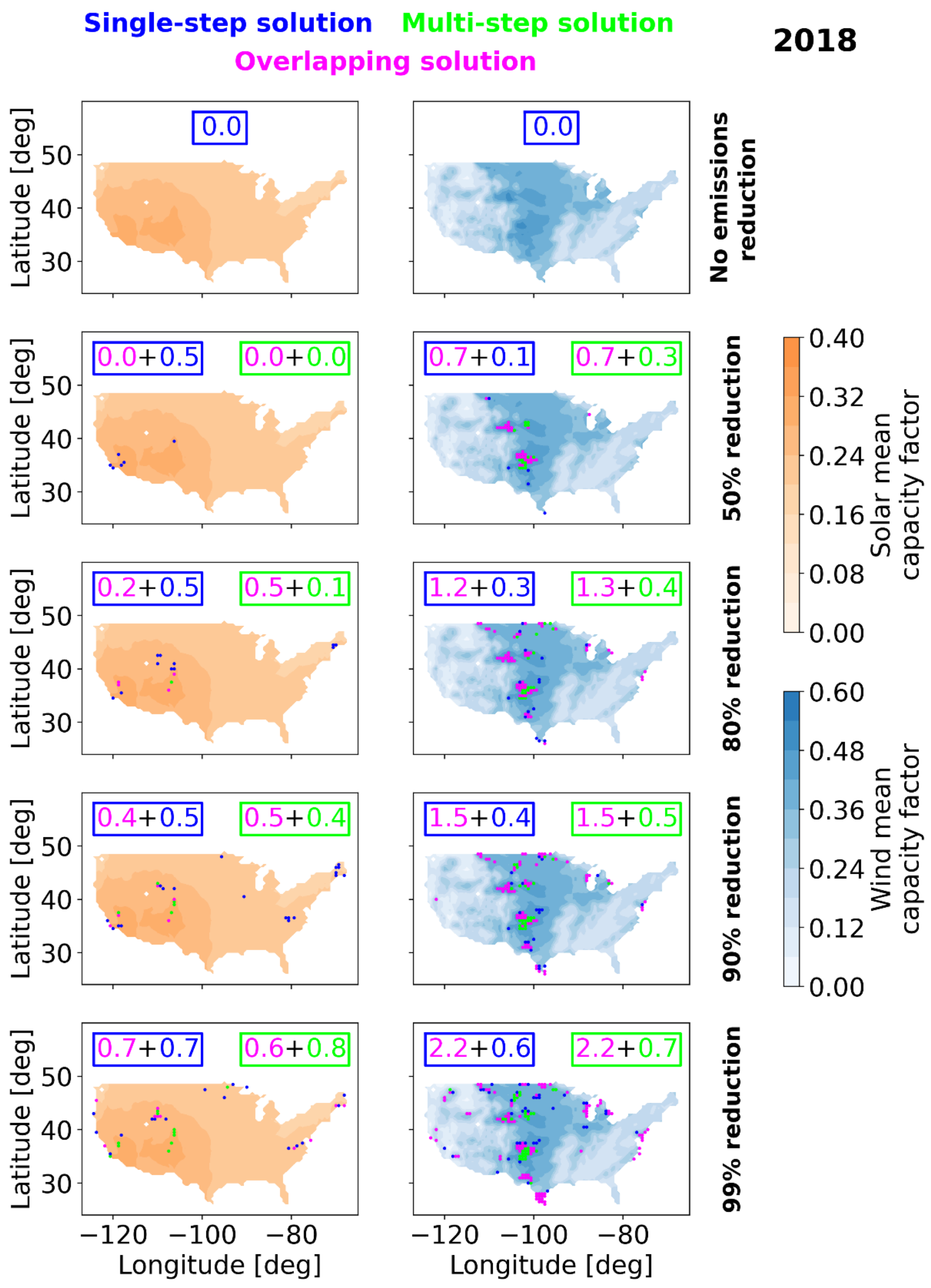

Figure S13. Locations of the wind and solar installations selected by our optimizer for the different emissions reduction cases for CONUS in the year 2018 when considering wind, solar, natural gas, and battery technologies. This figure is related to Fig. 3. For each map, we show the spatial distribution of the wind or solar mean capacity factor along with dots indicating the locations where generation capacity was installed in the multi-step and single-step least-cost solutions.

		Natural gas	Wind	Solar	Battery
		Power capacity	Power capacity	Power capacity	Energy capacity
**0% reduction**	Single-step	1.37	0.04	0.14	0.00
**50% reduction**	Single-step	0.99	0.79	0.45	0.00
	Multi-step	1.37	0.96	0.14	0.00
**80% reduction**	Single-step	0.79	1.51	0.62	0.00
	Multi-step	1.37	1.63	0.46	0.00
**90% reduction**	Single-step	0.63	1.86	0.87	0.00
	Multi-step	1.37	1.92	0.77	0.00
**99% reduction**	Single-step	0.25	2.52	1.39	0.41
	Multi-step	1.37	2.64	1.37	0.24

Table S1. Installed capacity for CONUS in the year 2019 when considering wind, solar, natural gas, and battery technologies. This table is related to Fig. 1. Values are normalized by mean electricity demand, which is approximately equal to 460 GW.

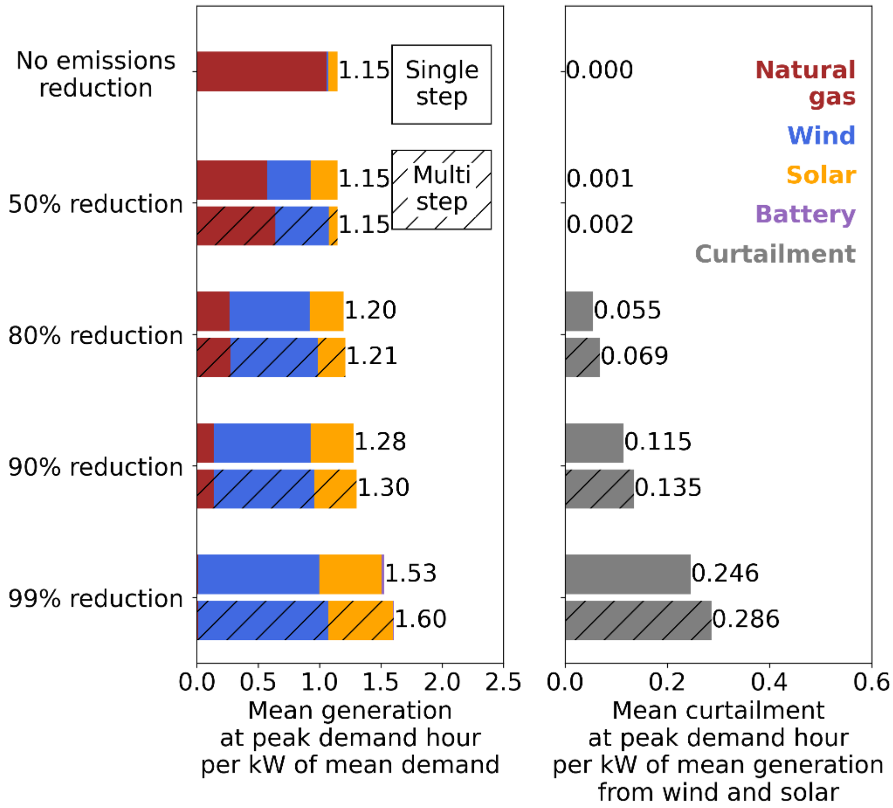

Figure S14. Mean generation and mean curtailment at peak demand hours for increasingly strict carbon emissions limits resulting from both multi-step and single-step optimizations for CONUS in the year 2019 when considering wind, solar, natural gas, and battery technologies. This figure is related to Fig. 1. The mean electricity demand is approximately equal to 460 GW. The procedure to calculate the mean generation at peak demand hours is the following: for each day of the year, select the peak demand hour, extract the generation mix, and normalize the generation mix with the mean demand; then average the normalized generation mix at peak demand hour over the whole year.

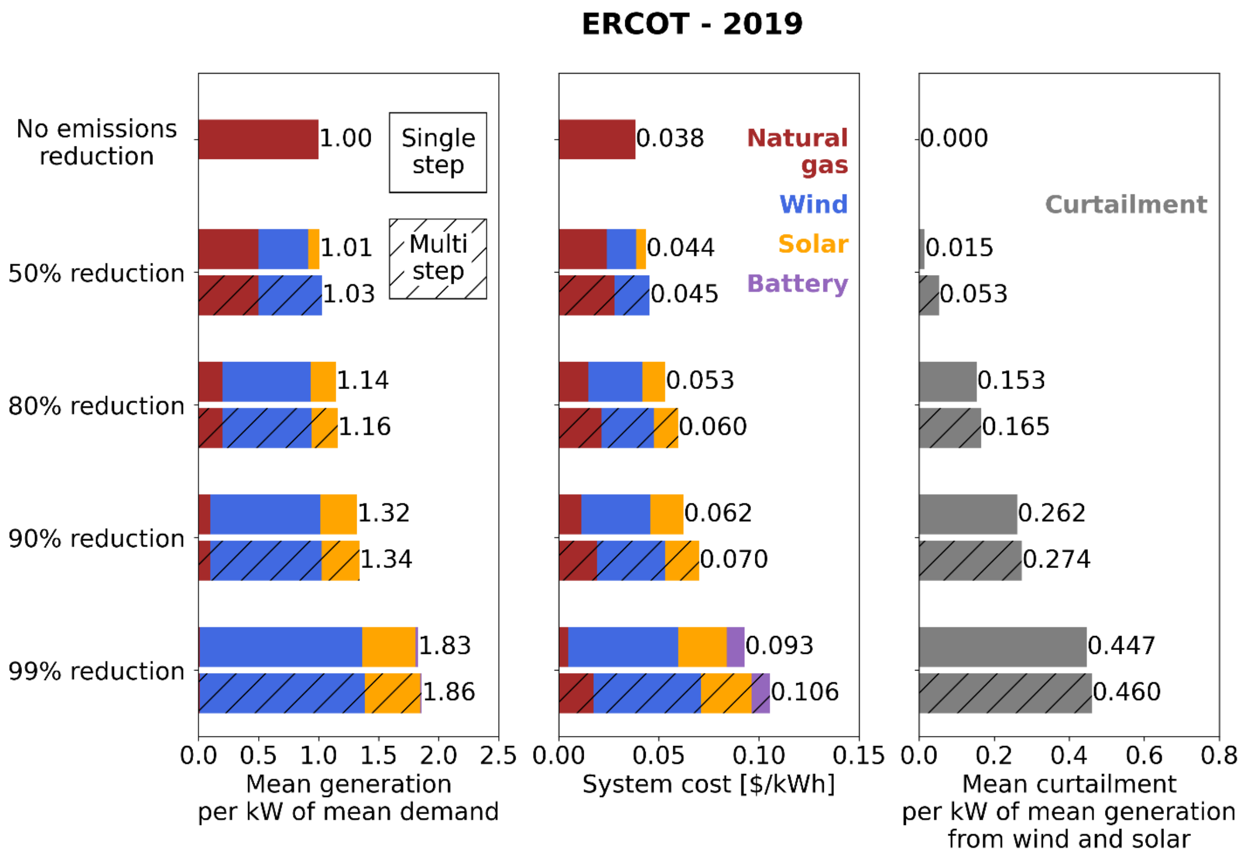

Figure S15. Mean generation, system level cost, and mean curtailment for increasingly strict carbon emissions limits resulting from both multi-step and single-step optimizations for ERCOT in the year 2019 when considering wind, solar, natural gas, and battery technologies. This figure is related to Fig. 1. The mean electricity demand is approximately equal to 44 GW.

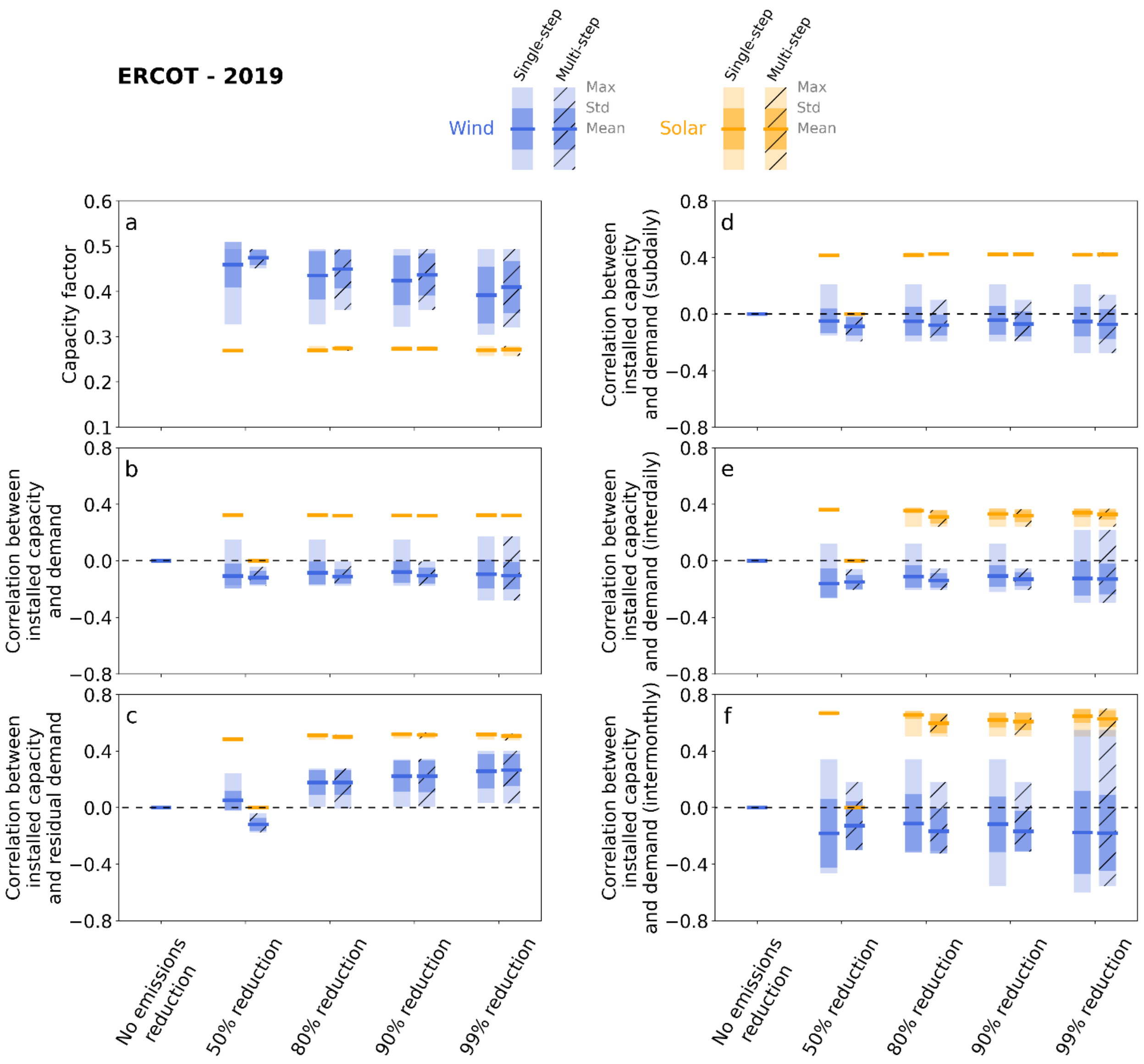

Figure S16. Statistical analysis of the wind and solar capacity factors of the optimized locations for various emission reduction targets for ERCOT in the year 2019 when considering wind, solar, natural gas, and battery technologies. This figure is related to Fig. 2. Panel a shows the mean and standard deviation of capacity factors of the chosen locations. Panel b shows the mean and standard deviation of correlation of wind and solar capacity factor time series with the demand time series. Panel c shows the mean and standard deviation of correlation of wind and solar capacity factor time series with the residual demand time series. Panels d, e, and f show the mean and standard deviation of correlations between wind and solar capacity factor time series and the demand time series after a filter has been applied.

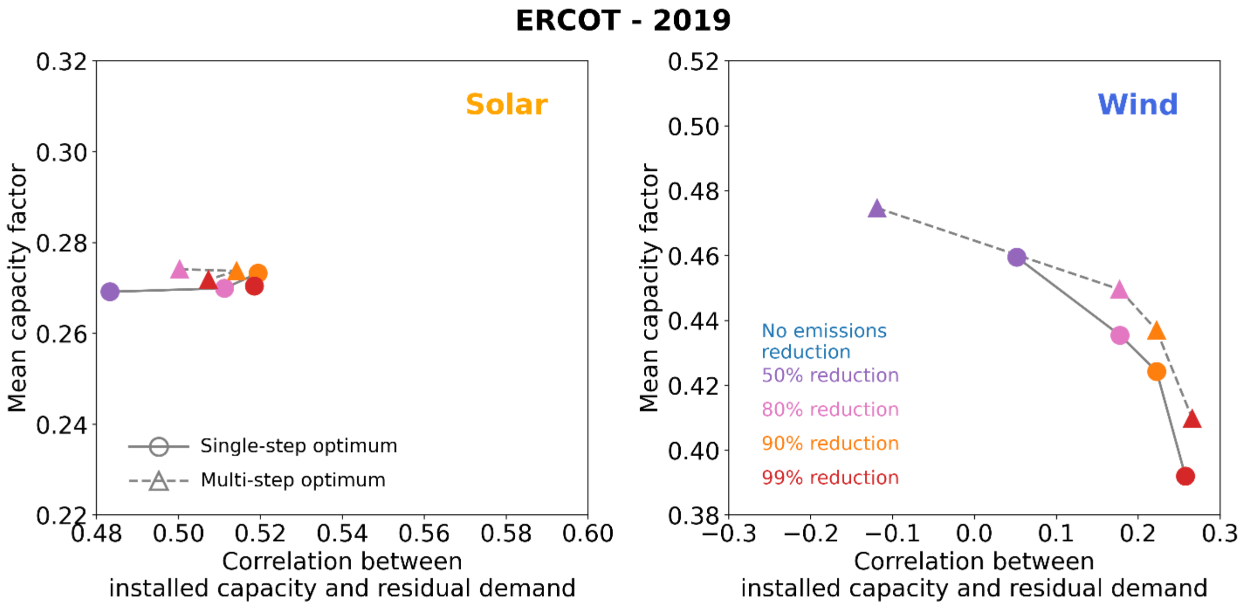

Figure S17. Mean capacity factors of wind and solar installations versus the correlation between the capacity factor time series and the residual demand for different emissions reduction targets for ERCOT in the year 2019 when considering wind, solar, natural gas, and battery technologies. This figure is related to Fig. 2. The left panel shows results for the solar installations, while the right panel for wind. For increasingly strict carbon emissions limits, mean capacity factors generally decrease and correlation with residual demand increase. If no marker is shown for a given emissions reduction target, it means that there is no built capacity, or the solution fully coincides with the stricter emissions reduction target (markers overlap).

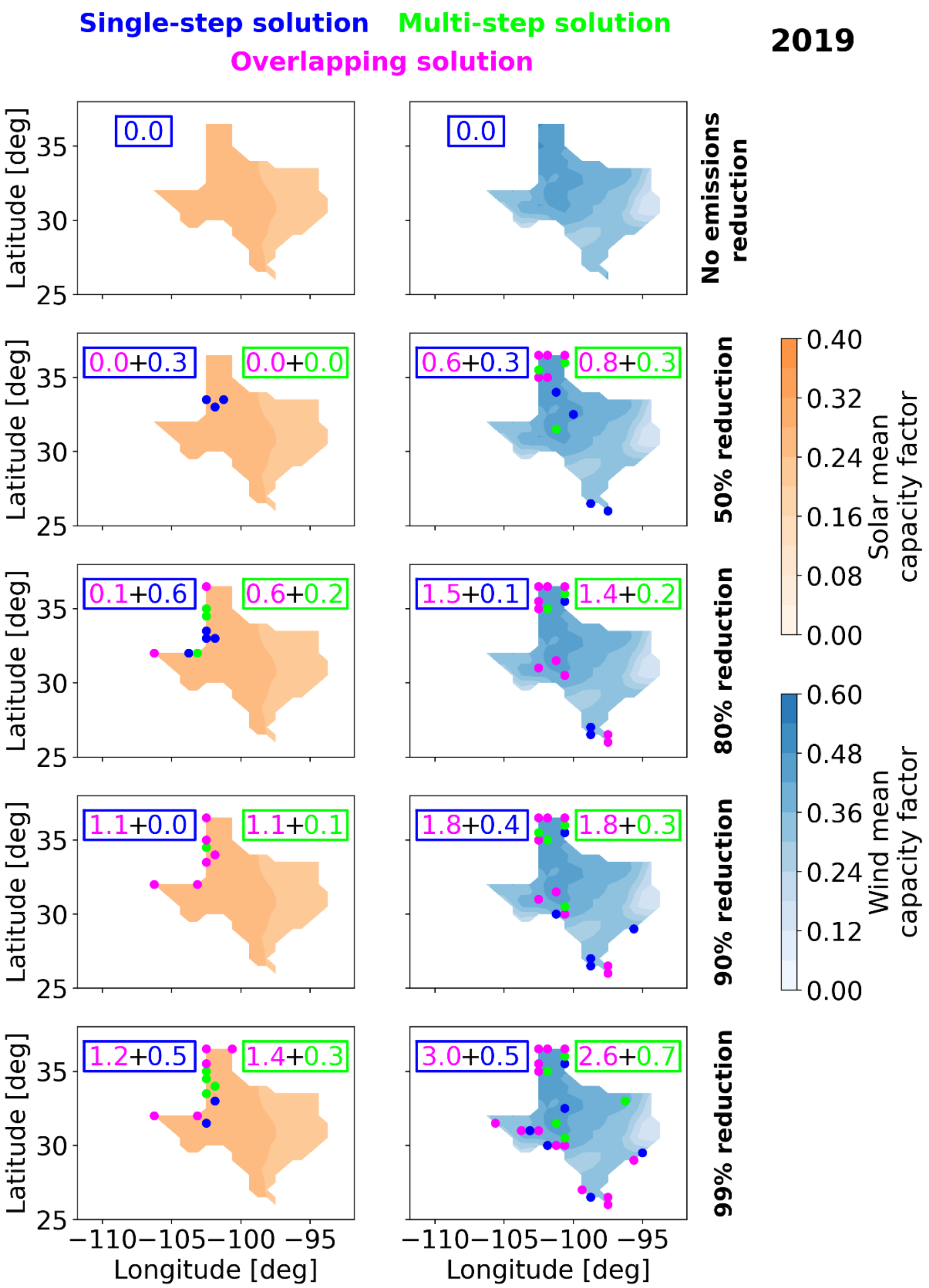

Figure S18. Locations of the wind and solar installations selected by our optimizer for the different emissions reduction cases for ERCOT in the year 2019 when considering wind, solar, natural gas, and battery technologies. This figure is related to Fig. 3. For each map, we show the spatial distribution of the wind or solar mean capacity factor along with dots indicating the locations where generation capacity was installed in the multi-step and single-step least-cost solutions.

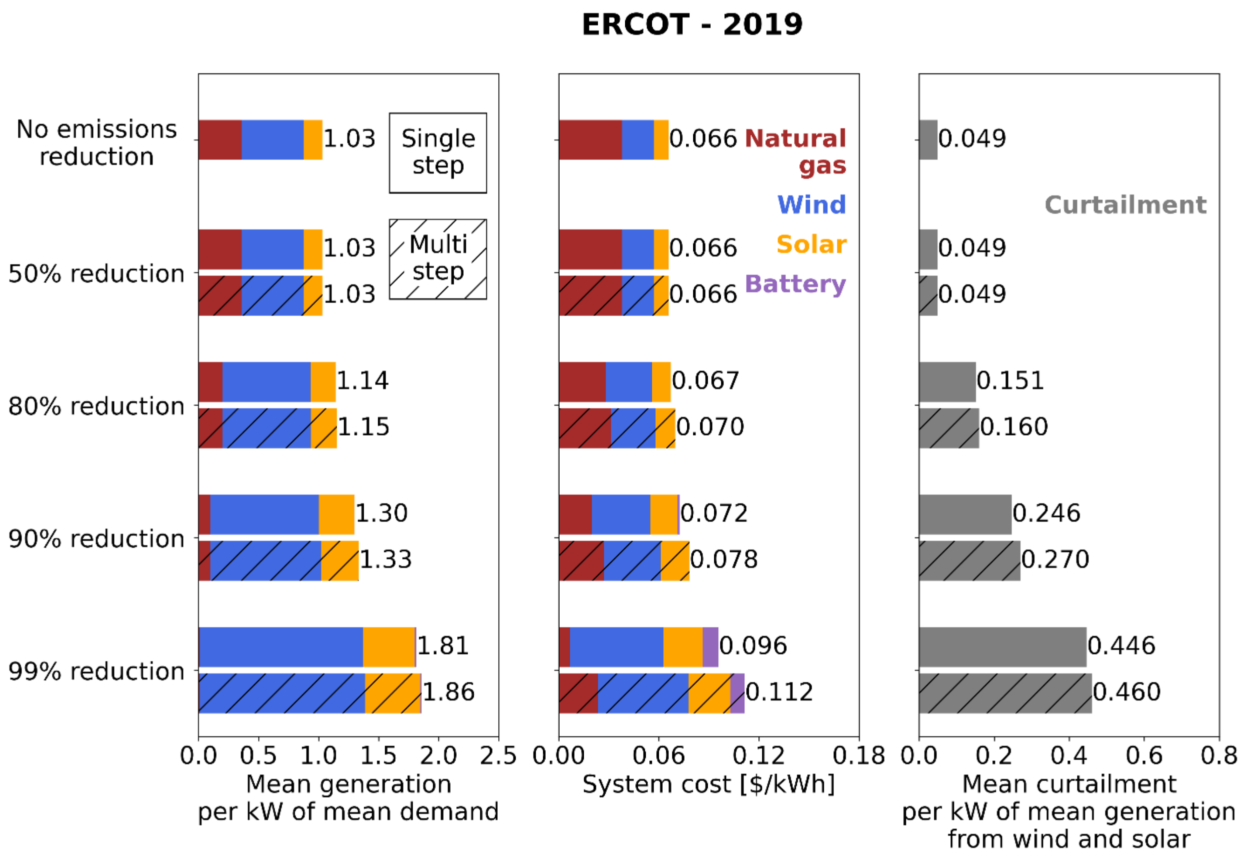

Figure S19. Mean generation, system level cost, and mean curtailment for increasingly strict carbon emissions limits resulting from both multi-step and single-step optimizations for ERCOT in the year 2019 when considering wind, solar, natural gas with 2x baseline cost, and battery technologies. This figure is related to Fig. 1. The mean electricity demand is approximately equal to 44 GW. Fixed and variable costs for natural gas are twice as much as the baseline costs reported in Tab. 1

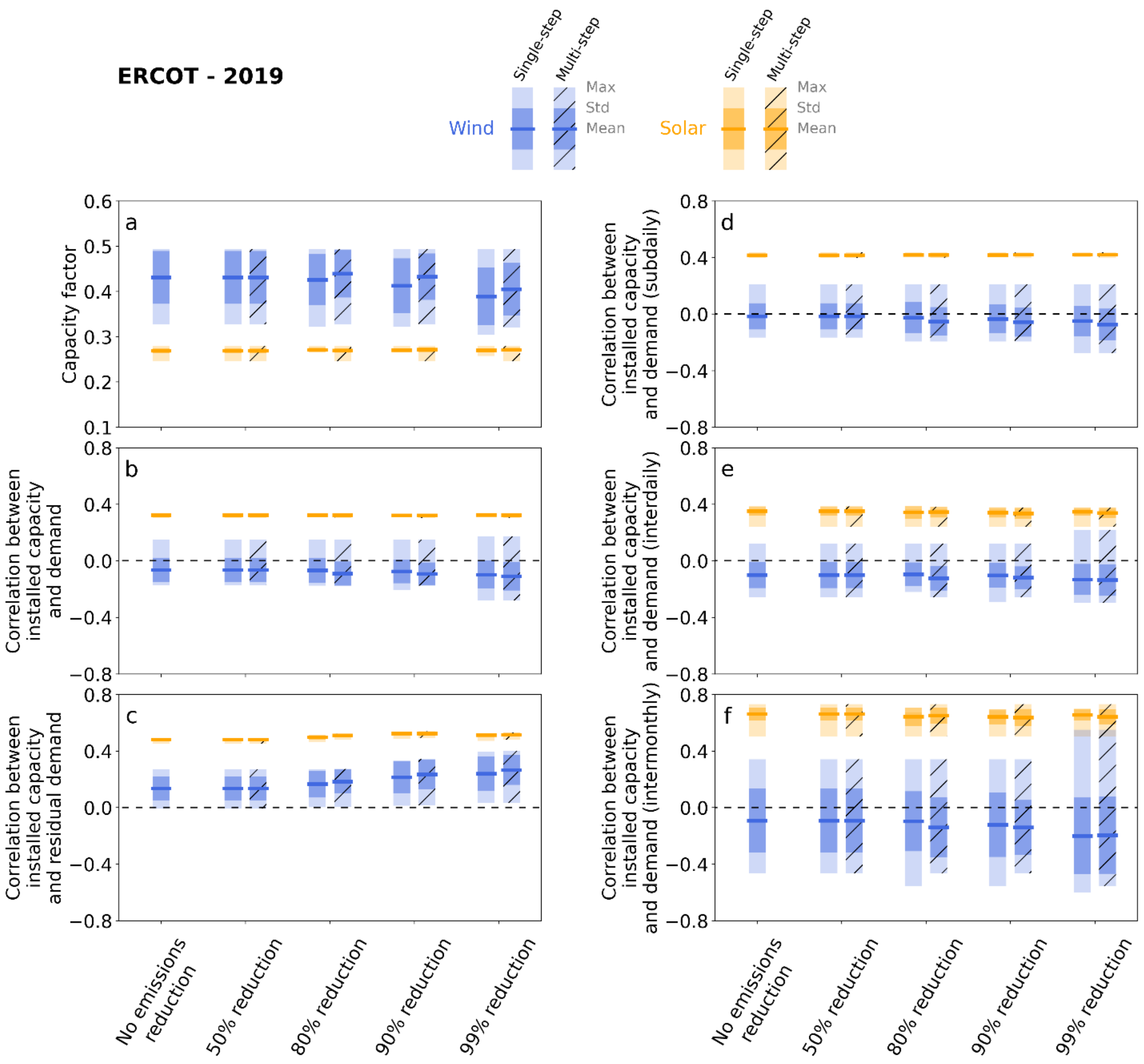

Figure S20. Statistical analysis of the wind and solar capacity factors of the optimized locations for various emission reduction targets for ERCOT in the year 2019 when considering wind, solar, natural gas with 2x baseline cost, and battery technologies. This figure is related to Fig. 2. Panel a shows the mean and standard deviation of capacity factors of the chosen locations. Panel b shows the mean and standard deviation of correlation of wind and solar capacity factor time series with the demand time series. Panel c shows the mean and standard deviation of correlation of wind and solar capacity factor time series with the residual demand time series. Panels d, e, and f show the mean and standard deviation of correlations between wind and solar capacity factor time series and the demand time series after a filter has been applied.

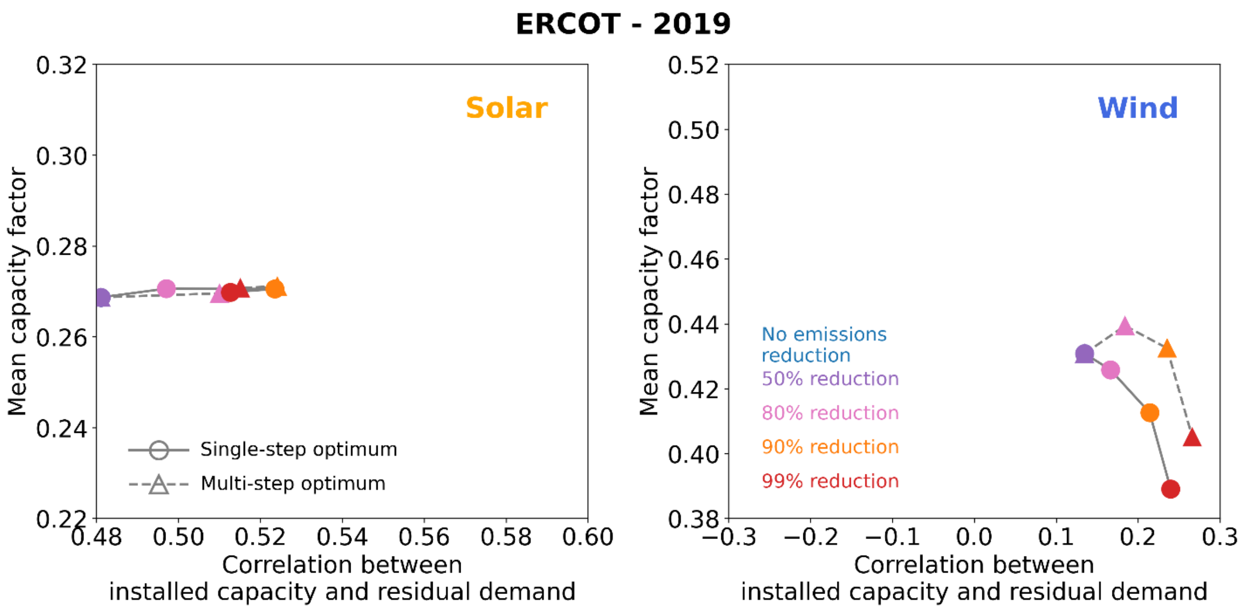

Figure S21. Mean capacity factors of wind and solar installations versus the correlation between the capacity factor time series and the residual demand for different emissions reduction targets for ERCOT in the year 2019 when considering wind, solar, natural gas with 2x baseline cost, and battery technologies. This figure is related to Fig. 2. The left panel shows results for the solar installations, while the right panel for wind. For increasingly strict carbon emissions limits, mean capacity factors generally decrease and correlation with residual demand increase. If no marker is shown for a given emissions reduction target, it means that there is no built capacity, or the solution fully coincides with the stricter emissions reduction target (markers overlap).

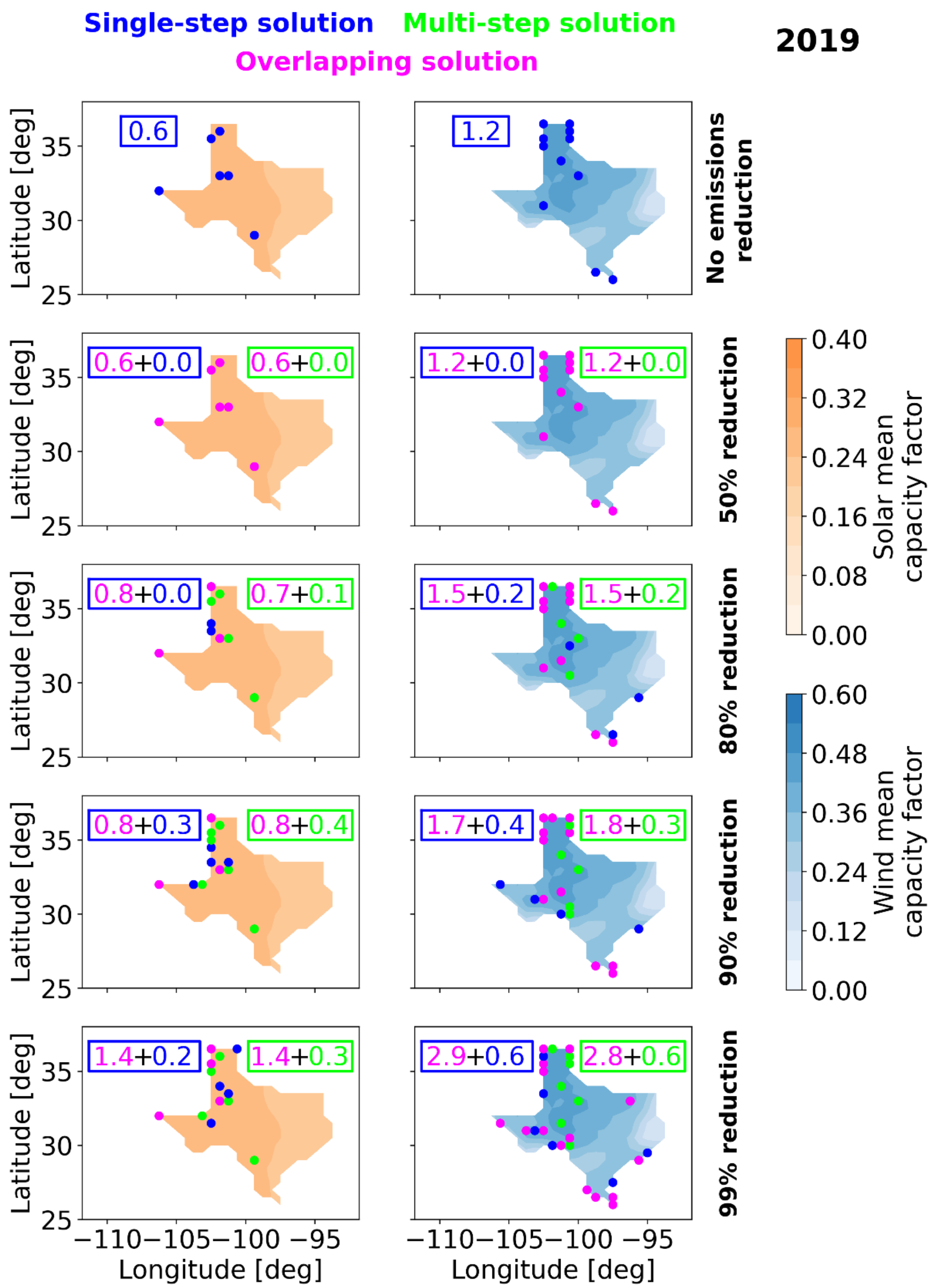

Figure S22. Locations of the wind and solar installations selected by our optimizer for the different emissions reduction cases for ERCOT in the year 2019 when considering wind, solar, natural gas with 2x baseline cost, and battery technologies. This figure is related to Fig. 3. For each map, we show the spatial distribution of the wind or solar mean capacity factor along with dots indicating the locations where generation capacity was installed in the multi-step and single-step least-cost solutions.

	**Nuclear**	**PGP storage**	**PGP electrolyzer + compressor**	**PGP fuel cell**
Capacity (fixed) cost type	Power capacity [$/kW]	Energy capacity [$/kWh]	Power capacity [$/kW]	Power capacity [$/kW]
Capacity (fixed) cost	6,317	0.21	1,918	4,968
Project life [yrs]	40	30	12.5	20
Discount rate [%]	7	7	7	7
Capital recovery factor [%]	7.5	8.06	12.26	9.44
Fixed O&M cost [$/kW-yr or [$/kWh-yr]	121.1	0.016	60.1	43.2
Variable O&M cost [$/kWh]				
Fuel cost [$/kWh]				
Efficiency			0.7	0.7
Self-discharge rate [% per hour]		0.00000001		
**Fixed cost [$/kW/h]**	**0.0679**	**0.00000373**	**0.0346**	**0.0585**
**Variable cost [$/kWh/h]**		**0.000001**	**0.000001**	

Table S2. Economic and cost assumptions when considering wind, solar, natural gas, battery, nuclear and PGP technologies. This table is related to Tab. 1. Values taken from the U.S. Energy Information Administration and the National Renewable Energy Laboratory.

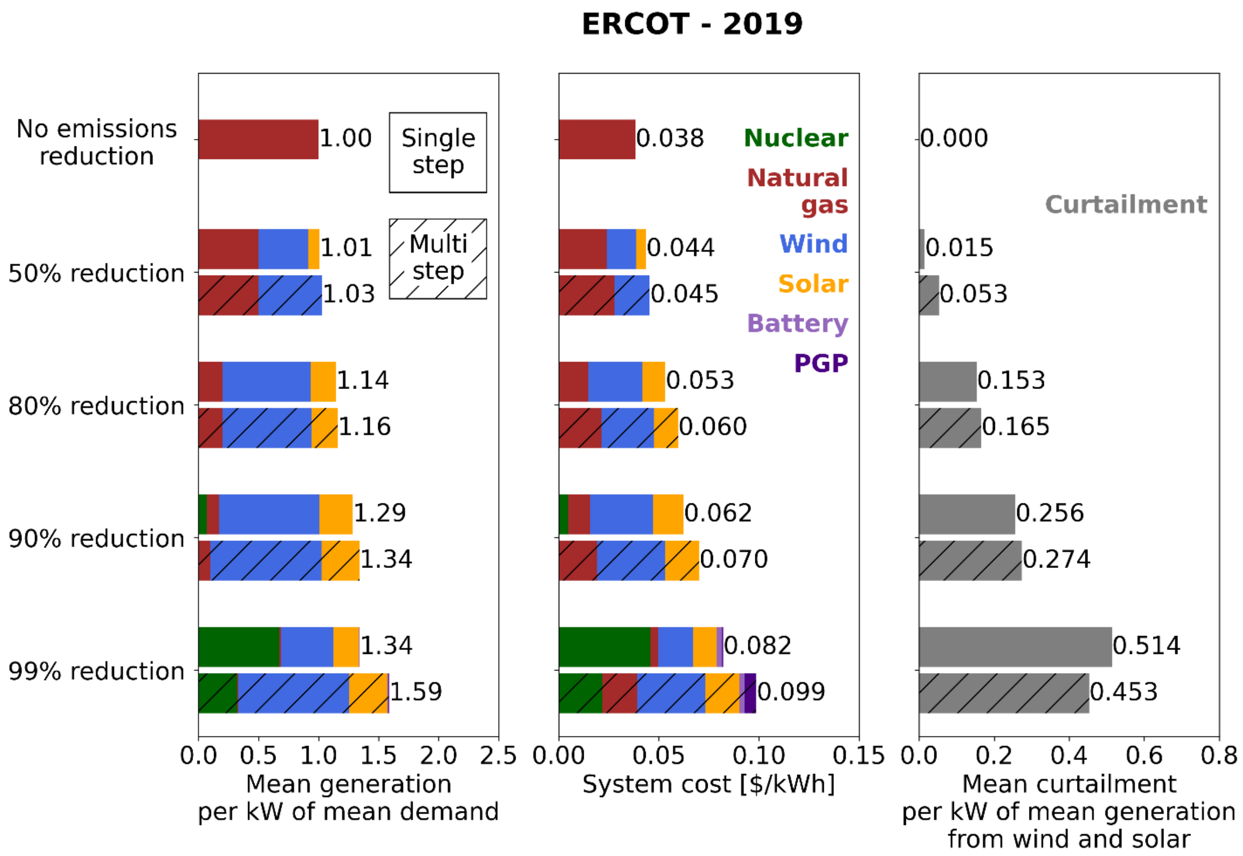

Figure S23. Mean generation, system level cost, and mean curtailment for increasingly strict carbon emissions limits resulting from both multi-step and single-step optimizations for ERCOT in the year 2019 when considering wind, solar, natural gas, battery, nuclear and PGP technologies. This figure is related to Fig. 1. The mean electricity demand is approximately equal to 44 GW.

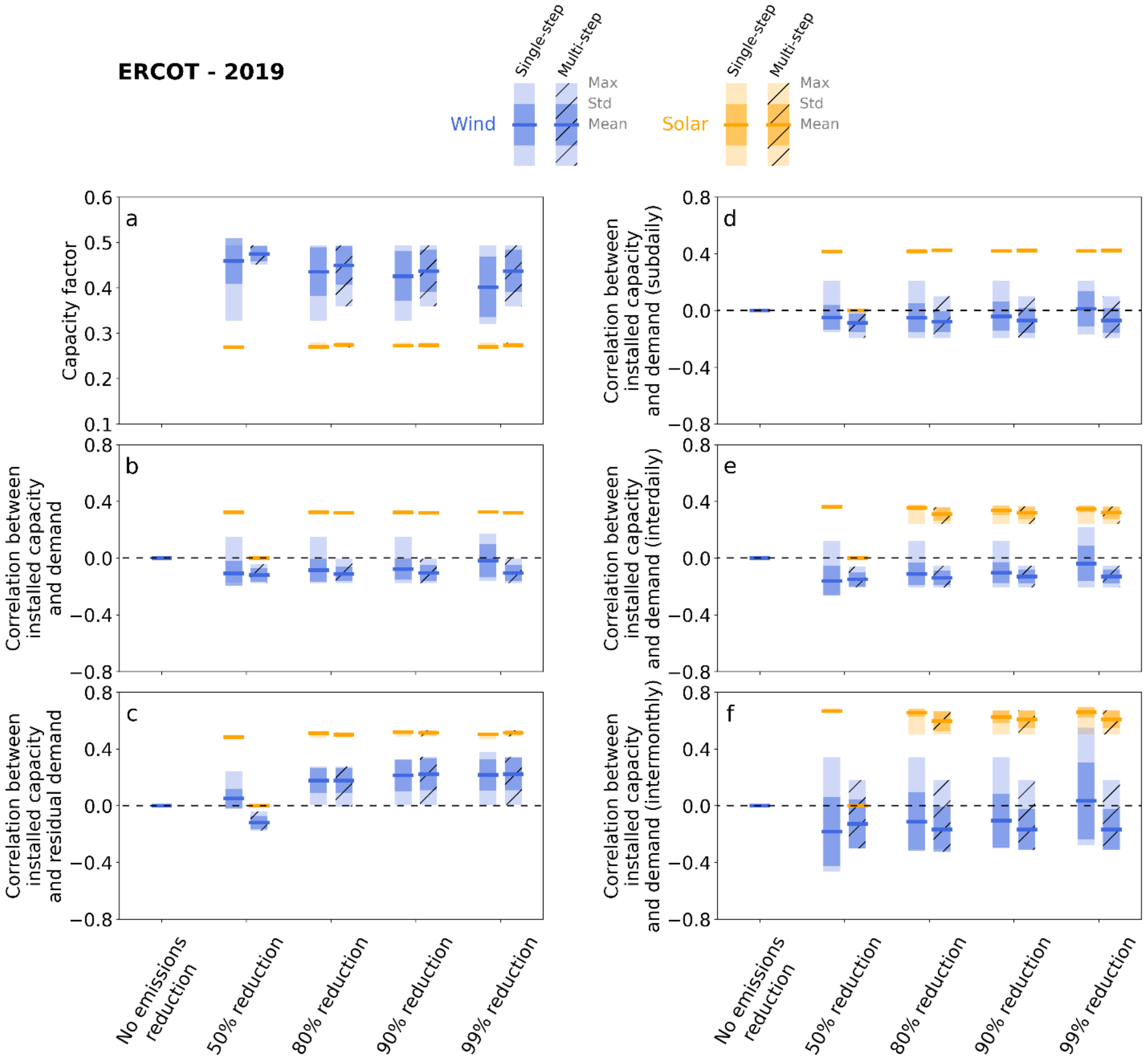

Figure S24. Statistical analysis of the wind and solar capacity factors of the optimized locations for various emission reduction targets for ERCOT in the year 2019 when considering wind, solar, natural gas, battery, nuclear and PGP technologies. This figure is related to Fig. 2. Panel a shows the mean and standard deviation of capacity factors of the chosen locations. Panel b shows the mean and standard deviation of correlation of wind and solar capacity factor time series with the demand time series. Panel c shows the mean and standard deviation of correlation of wind and solar capacity factor time series with the residual demand time series. Panels d, e, and f show the mean and standard deviation of correlations between wind and solar capacity factor time series and the demand time series after a filter has been applied.

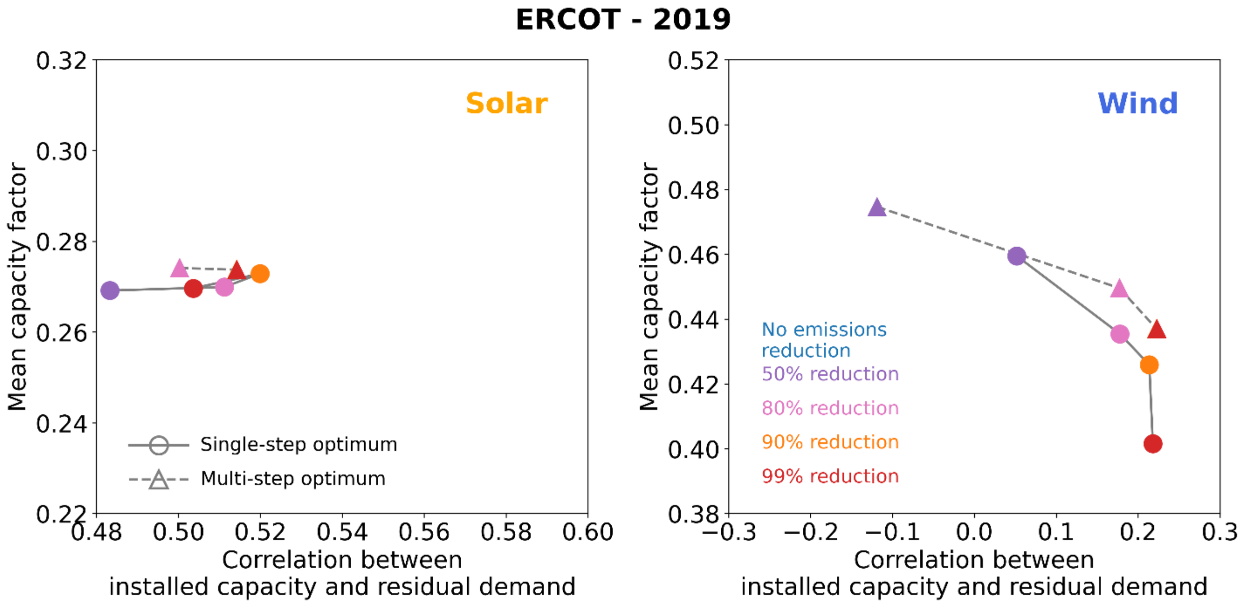

Figure S25. Mean capacity factors of wind and solar installations versus the correlation between the capacity factor time series and the residual demand for different emissions reduction targets for ERCOT in the year 2019 when considering wind, solar, natural gas, battery, nuclear and PGP technologies. This figure is related to Fig. 2. The left panel shows results for the solar installations, while the right panel for wind. For increasingly strict carbon emissions limits, mean capacity factors generally decrease and correlation with residual demand increase. If no marker is shown for a given emissions reduction target, it means that there is no built capacity, or the solution fully coincides with the stricter emissions reduction target (markers overlap).

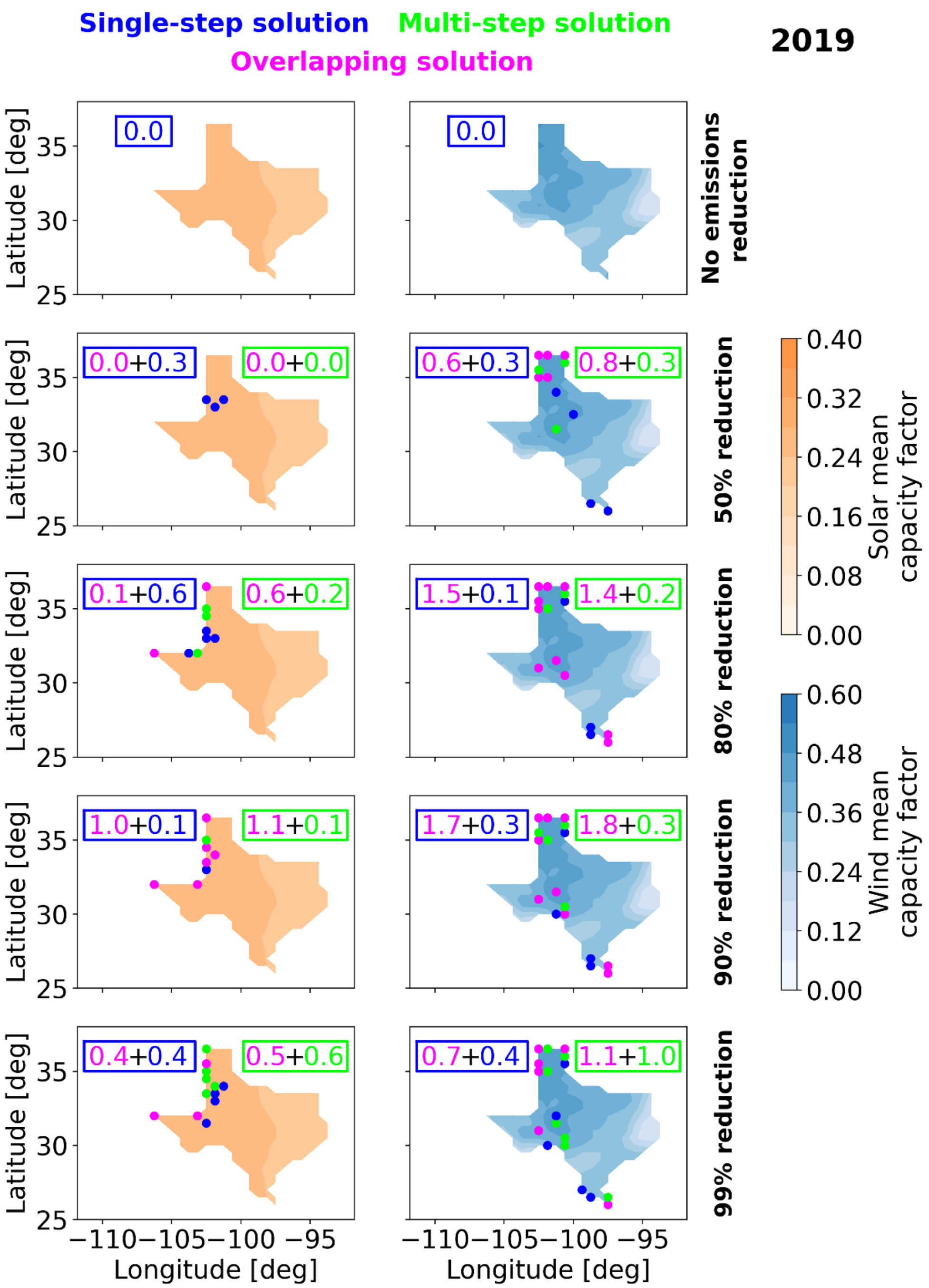

Figure S26. Locations of the wind and solar installations selected by our optimizer for the different emissions reduction cases for ERCOT in the year 2019 when considering wind, solar, natural gas, battery, nuclear and PGP technologies. This figure is related to Fig. 3. For each map, we show the spatial distribution of the wind or solar mean capacity factor along with dots indicating the locations where generation capacity was installed in the multi-step and single-step least-cost solutions.
